# Chemical Profiling and Cheminformatic Insights into *Piper* Essential Oils as Sustainable Antimicrobial Agents Against Pathogens of Cocoa Crops

**DOI:** 10.3390/molecules31020326

**Published:** 2026-01-17

**Authors:** Diannefair Duarte, Marcial Fuentes-Estrada, Yorladys Martínez Aroca, Paloma Sendoya-Gutiérrez, Manuel I. Osorio, Osvaldo Yáñez, Carlos Areche, Elena Stashenko, Olimpo García-Beltrán

**Affiliations:** 1 Facultad de Ciencias Agrarias, Universidad de Pamplona, Carretera a Bucaramanga 1 Km, Pamplona 543050, Colombia; 2 Institución Educativa Otoniel Guzmán, Secretaría de Educación del Tolima, Venadillo 730580, Colombia; 3 Facultad de Ciencias, Ingeniería e Innovación, Universidad de Ibagué, Carrera 22 Calle 67, Ibagué 730002, Colombia; 4 Facultad de Odontología, Universidad Andres Bello, Santiago 8370133, Chile; 5 Facultad de Medicina, Universidad Diego Portales, Santiago 8370007, Chile; 6 Centro de Modelación Ambiental y Dinámica de Sistemas (CEMADIS), Facultad de Ingeniería y Negocios, Universidad de Las Américas, Santiago 7500975, Chile; 7 Departamento de Química, Facultad de Ciencias, Universidad de Chile, Casilla 653, Santiago 3521000, Chile; 8 Research Center of Excellence CENIVAM, CIBIMOL, Universidad Industrial de Santander, Building 45, UIS, Carrera 27, Calle 9, Bucaramanga 680002, Colombia; 9 Centro de Estudios e Investigación en Salud y Sociedad (EISS), Universidad Bernardo O’Higgins, General Gana 1702, Santiago 8370854, Chile; 10 Colaboratorio de Investigación en Bioeconomía Regional, Universidad de Ibagué, Carrera 22 Calle 67, Ibagué 730002, Colombia

**Keywords:** *Theobroma cacao*, essential oil, *Piper*, antimicrobial, moniliasis, phytophthora

## Abstract

This study evaluates the chemical profile and antifungal efficacy of essential oils from *Piper glabratum*, *Piper friedrichsthalii*, and *Piper cumanense* against the cocoa pathogens *Moniliophthora roreri* and *Phytophthora palmivora*. Microwave-assisted hydrodistillation followed by GC-MS analysis identified 80 constituents, predominantly monoterpenes and sesquiterpenes, which exhibited significant mycelial inhibition comparable to commercial fungicides. Beyond basic characterization, a comprehensive chemoinformatic analysis was conducted to elucidate the molecular mechanisms driving this bioactivity. The computed physicochemical landscape reveals a dominant lipophilic profile (average LogP 3.4) and low polarity (TPSA 11.5 Å^2^), characteristics essential for effective fungal membrane penetration. Structural mining identified conserved benzene and cyclohexene scaffolds alongside specific 1,3-benzodioxole moieties, while Maximum Common Substructure (MCS) analysis uncovered high similarity clusters among phenylpropanoids and sesquiterpenes. These findings suggest a synergistic mode of action where conserved structural backbones and interchangeable diastereomers facilitate membrane destabilization and ion leakage. Consequently, the integrative chemoinformatic profiling elucidates the molecular basis of this efficacy, positioning these *Piper* essential oils not merely as empirical alternatives, but as sources of rationally defined synergistic scaffolds for next-generation sustainable fungicides.

## 1. Introduction

**Figure 1:**
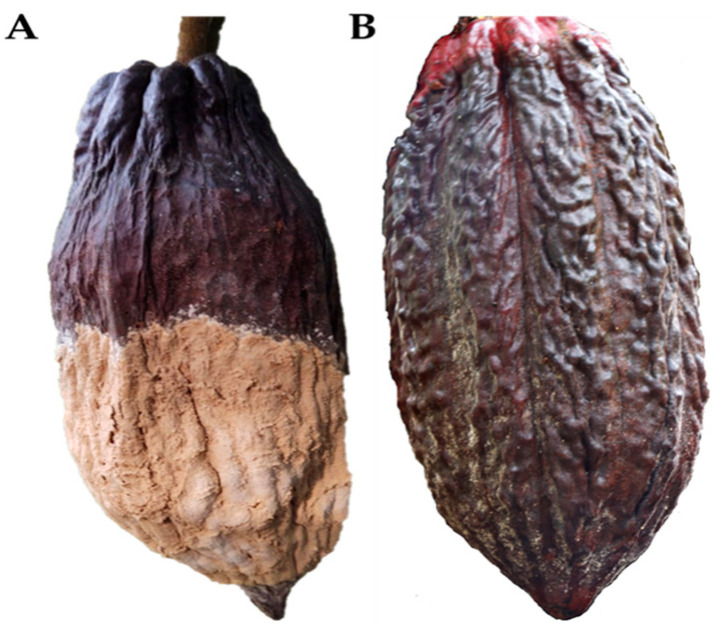
(**A**) Fruit of *Theobroma cacao* affected by the fungus *Moniliophthora roreri*; (**B**) Fruit of *Theobroma cacao* affected by the Oomycota *Phytophthora palmivora*.

Cocoa (*Theobroma cacao* L.) is an ancestral plant [1] of great cultural, ecological, and economic importance. Belonging to the Malvaceae family, beans are generally classified into three types: Criollo, Forastero, and Trinitario, which have distinct physical, chemical, and functional properties. Dried seeds are sold separately from the fruit, and their aromatic and compositional quality is determined by factors such as origin, processing, and the effect of soil and climate conditions [2,3]. Several studies indicate that cacao originated at the headwaters of the Amazon basin and that a natural population spread across the central part of the Amazon-Guiana region, westward and northward, constituting the group known as Forastero-Amazonian and a second group known as Criollo, the latter being well accepted in the market due to its high organoleptic qualities [4,5,6]. Cocoa is currently grown commercially in Asia and Oceania, Central and South America, and Africa. These regions account for 6%, 24%, and 70% of global production, respectively [7]. Since 2016, Colombia has increased its cultivation area as part of the “cocoa for peace” illicit crop substitution strategy promoted under the peace agreement [8]. Unfortunately, cultivation area expansion, combined with high humidity and advancing deforestation, has led to the emergence of several natural enemies, such as the fungi *Colletrotrichum gloesporioides* and *Moniliophthora roreri* and the oomycete *Phytophthora palmivora* [9,10] and other factors that affect the decline in production of this crop, resulting in significant economic loss for countries that devote large areas of land to its production [11,12]. For this reason, these diseases are considered the main phytosanitary problem affecting cocoa in the Americas. *M. roreri* and *P. palmivora* (Figure 1) are the pathogens responsible for the diseases causing the most significant crop losses in cocoa cultivation in Colombia. Specifically, moniliasis is a specialized fungus that affects cocoa pods; in Colombia, losses of up to 40% and 100% have been recorded [13]. It has a high survival rate in various environments, characterized by rapid growth and spread. In general, commercial genotypes are highly susceptible to this pathogen [14], and current disease control methods are ineffective, increasing production costs [13].

Currently, various strategies are employed to control the proliferation of these microorganisms, including social, biological, genetic, and chemical control. Chemical control uses substances that can be toxic for humans and the environment due to their content of copper oxychloride, copper hydroxide, copper (II) sulfate pentahydrate, cuprous oxide, tribasic copper sulfate monohydrate, and organic compounds [15] such as metalaxyl [16], However, the use of synthetic chemical fungicides, despite their proven effectiveness in controlling the pathogen, results in production costs that make profitability unfeasible for the producer, and the environmental damage they cause has a significant impact. Additionally, more rudimentary practices, such as phytosanitary pruning, are also employed during specific periods. It requires cultural management primarily focused on reducing tree size, along with continuous pruning and sanitation, efficient, timely appropriate collection and disposal of diseased fruit, as well as effective shade management [17].

The use of active ingredients and essential oils of plant origin is a priority strategy for the sustainable control of diseases in crops of high economic value, such as cocoa, due to their antifungal and antibacterial activity and their ability to modulate induced resistance in plants. Unlike synthetic fungicides, these active ingredients act by interfering with the cellular integrity of phytopathogens, inhibiting spore germination, and even altering signaling pathways related to virulence and quorum sensing [18]. The *Piper* genus represents one of the most promising sources of essential oils and bioactive metabolites for the natural control of diseases in strategic crops, such as cocoa, due to its high phytochemical diversity and proven antifungal and antibacterial activity [19,20,21,22]. This genus comprises nearly 2000 species, widely distributed in tropical ecosystems throughout Latin America [23].

Additionally, a comprehensive chemoinformatics analysis was conducted to elucidate the molecular properties underlying the observed biological activities. This includes the calculation of physicochemical descriptors, molecular similarity analysis using Tanimoto coefficients, scaffold identification [24], and principal component analysis (PCA) to establish structure-activity relationships [25]. This integrated approach, combining traditional phytochemical analysis with modern computational chemistry, provides a robust framework for understanding the antimicrobial mechanisms and optimizing the potential of these natural products as sustainable alternatives to synthetic fungicides in cocoa disease management. Finally, this work is essential in cocoa-producing countries, as it seeks sustainable alternatives based on natural ingredients through the use of active compounds, and develops new knowledge that allows for control over the development of diseases such as moniliasis and phytophthora through the use of essential oils.

## 2. Results and Discussion

### 2.1. Identification of the Active Principles of the Essential Oils of the Species P. glabatrum, P. friedrichsthalii, and P. cumanense

In this study, essential oils were extracted from wild plants that have no economic value, showing different yields based on fresh plant material. Thus, a yield of 0.83% was obtained from the species *P. glabatrum*, 0.60% from *P. Friedrichsthalii*, and 0.56% from *P. cumanense*. Although the yields are below 1%, some species show promise for the development of bioproducts, the use of microwave-assisted hydrodistillation (MWHD) offers a fast and energy-efficient alternative for extracting essential oils, using selective microwave heating to break down plant tissue and enhance the release of volatile compounds. This typically results in higher yields and better preservation of thermolabile components compared to conventional hydrodistillation. However, MWHD presents some challenges, such as the need for careful optimization to avoid uneven heating and its scalability. Despite these limitations, MWHD represents a valuable green extraction technology. A total of eighty compounds were identified in the essential oils of *P. glabatrum*, *P. friedrichsthalii*, and *P. cumanense* (Table 1). The constituents were eluted using two columns (DB-5MS and DB-WAX), and the results were compared individually, selecting those with relatively abundant amounts greater than 0.1% using the DB-5MS column. These eighty compounds were grouped into straight-chain hydrocarbons, alcohols, monoterpenes, diterpenes, coumarins, allylbenzenes, alkylbenzenes, and phenylpropenes (Table 1).

The main compounds in the species *P. glabratum* (Table 1; Figure S1; Supplementary Materials) were α-pinene (16.6%), 1,8-cineol (22.2%), linalool (10.8%), *trans*-β-caryophyllene (7.2%) and germacrene D (5.8%); *P. friedrichsthalii* (Table 1, Figure S2; Supplementary Materials) the most abundant compounds were α-terpinene (11.2%), γ-terpinene (15.7%), *p*-cimene (5.1%), terpinolene (5.0%) *trans*-β-caryophyllene (7.1%), germacrene D (6.1%), 2-*cis*-6-*trans*-farnesol (13.1). The major constituents in the oil of *P. cumanense* (Table 1; Figure S3; Supplementary Materials) were apiole (36.3%), terpinen-4-ol (6.4%), *trans*-β-caryophyllene (14.4%), germacrene D (4.3%), and viridiflorol (5.3%). These results were compared with those obtained using the DB-WAX column, showing the same major compounds and concentration ratios, as well as a chromatogram consistent with that obtained using the DB-5MS column.

For the statistical analysis, a general descriptive analysis was conducted for each of the treatments (Tables S1–S3; Supplementary Materials) to determine the significance of the growth diameter means [26]. For the development of the assay, a design was used with five concentrations of essential oils of *P. glabratum*, *P. friedrichsthalii*, and *P. cumanense*, numbered from T1 to T5, a negative control T6 without treatment, and a positive control T7, a synthetic fungicide such as copper oxychloride (Cu_2_(OH)_3_Cl), for the assay against *M. roreri*. For the T7 assay against *P. palmivora*, metalaxyl was used as the antimicrobial product.

**Table 1:** GC/MS characterization of the essential oil obtained by steam distillation of the leaves of *P. glabratum*, *P. friedrichsthalii*, and *P. cumanense*.

No	Compound	Linear Retention Indices				% Area, GC-FID DB-5MS			Metabolite Type	Criteria for Identificatio
		DB-5MS		DB-WAX		*P. glabratum*	*P. friedrichsthalii*	*P. cumanense*		
		Exp.	Lit.	Exp.	Lit.					
1	*cis*-Hex-3-en-1-ol	856	850 [27]	1384	1380 [28]	0.5	1.0	1.0	Aliphatic alcohol	a, b
2	*trans*-Hex-2-en-1-ol	866	865 [28]	1408	1400 [28]	0.5	0.8	-	Aliphatic alcohol	a, b
3	Hexan-1-ol	869	870 [27]	1352	1351 [28]	0.4	0.4	0.5	Aliphatic alcohol	a, b
4	α-Thujene	928	930 [27]	1026	1027 [28]	0.1	0.2	1.0	Monoterpene	a, b
5	α-Pinene	938	932 [27]	1024	1025 [28]	16.6	2.4	0.8	Monoterpene	a, b, c
6	Camphene	953	954 [27]	1065	1069 [28]	0.5	0.1	4.0	Monoterpene	a, b, c
7	Sabinene	976	975 [27]	1122	1122 [28]	-	0.4	-	Monoterpene	a, b, c
8	β-Pinene	981	974 [29]	1110	1110 [28]	4.0	3.8	0.3	Monoterpene	a, b
9	β-Myrcene	990	990 [27]	1166	1161 [28]	1.2	0.9	0.6	Monoterpene	a, b, c
10	α-Phellandrene	1009	1002 [27]	1168	1168 [28]	4.1	0.3	-	Monoterpene	a, b, c
11	Δ^3^-Carene	1012	1011 [27]	1150	1147 [28]	-	1.3	-	Monoterpene	a, b
12	α-Terpinene	1019	1017 [27]	1182	1178 [28]	0.1	11.2	1.5	Monoterpene	a, b, c
13	*p*-Cimene	1028	1024 [27]	1275	1270 [28]	1.0	5.1	1.2	Aromatic monoterpene	a, b, c
14	Limonene	1033	1029 [27]	1202	1198 [28]	3.7	0.4	0.8	Monoterpene	a, b, c
15	β-Phellandreno	1035	1029 [27]	1211	1209 [28]	-	0.4	-	Monoterpene	a, b, c
16	*cis*-β-Ocimene	1037	1037 [27]	1234	1250 [28]	-	-	3.3	Monoterpene	a, b
17	1,8-Cineol	1039	1031 [27]	1214	1211 [28]	22.2	-	1.6	Oxygenated monoterpene	a, b, c
18	*trans*-β-Ocimene	1048	1050 [27]	1234	1250 [28]	-	-	3.8	Monoterpene	a, b
19	γ-Terpinene	1061	1059 [27]	1249	1245 [28]	0.4	15.7	3.8	Monoterpene	a, b, c
20	*cis*-Linalool oxide	1073	1075 [28]	1446	1446 [28]	0.5	-	-	Oxygenated monoterpene	a, b
21	*cis*-Sabinene hydrate	1074	1070 [27]	1468	1460 [28]	0.4	-	0.6	Oxygenated monoterpene	a, b
22	*trans*-Linalool oxide	1089	1083 [28]	1474	1454 [28]	0.5	-	-	Oxygenated monoterpene	a, b
23	Terpinolene	1089	1088 [27]	1286	1282 [28]	0.3	5.0	1.2	Monocyclic monoterpene	a, b
24	Fenchone	1093	1088 [28]	1403	1400 [28]	0.2	-	-	Oxygenated monoterpene	a, b
25	Linalool	1103	1096 [27]	1550	1543 [28]	10.8	-	2.8	Monoterpene	a, b, c
25	*trans*-Sabinene hydrate	1104	1098 [27]	1553	1549 [28]	-	-	0.4	Oxygenated monoterpene	a, b
27	2,2,6-Trimethyl-3-keto-6-vinyl-tetrahydropyran	1108	1108 [29]	1477	-	0.3	-		Oxygenated monoterpene	a, b
28	*trans*-*p*-Ment-2-en-1-ol	1129	1137 [28]	1567	1584 [28]	-	-	-	Oxygenated monoterpene	a, b
29	Camphor	1154	1146 [27]	1526	1515 [28]	0.4	-	3.6	Oxygenated monoterpene	a, b
30	Camphene hydrate	1162	1149 [27]	1602	1591 [29]	-	-	0.3	Oxygenated monoterpene	a, b
31	Isoborneol	1170	1157 [29]	1671	1660 [29]	-	-	1.9	Oxygenated monoterpene	a, b
32	δ-Terpineol	1175	1166 [29]	1675	1679 [28]	0.6	-	-	Oxygenated monoterpene	a, b
33	Borneol	1179	1169 [27]	1707	1700 [28]	0.2	-	0.3	Oxygenated monoterpene	a, b
34	Terpinen-4-ol	1186	1177 [27]	1604	1601 [28]	0.4	-	6.4	Oxygenated monoterpene	a, b, c
35	α-Terpineol	1201	1190 [28]	1702	1694 [28]	3.4	-	0.5	Oxygenated monoterpene	a, b, c
36	Piperitone	1260	1264 [29]	1732	1730 [28]	-	-	1.0	Monoterpene	a, b
37	Eugenol	1355	1356 [27]	2175	2163 [28]	-	2.1	-	Phenylpropanoid	a, b
38	Cyclosativene	1378	1374 [29]	1489	1483 [28]	0.4	-	-	Sesquiterpene	a, b
39	α-Copaene	1384	1376 [27]	1499	1491 [28]	2.5	0.3	0.9	Sesquiterpene	a, b
40	β-Elemene	1396	1390 [27]	1600	1591 [28]	0.6	1.3	1.1	Sesquiterpene	a, b
41	Methyleugenol	1401	1403 [27]	2018	2006 [28]	-	0.7	-	Phenylpropanoid	a, b
42	α-Gurjunene	1415	1409 [27]	1539	1529 [28]	-	-	0.7	Sesquiterpene	a, b
43	β-Elemene	1427	1420 [29]	1582	1576 [29]	0.8	-	-	Sesquiterpene	a, b
44	*trans*-β-Caryophyllene	1434	1427 [29]	1611	1599 [28]	7.2	7.1	14.7	Sesquiterpene	a, b, c
45	*trans*-α-Bergamotene	1440	1432 [27]	1594	1576 [28]	2.4	-	-	Sesquiterpene	a, b
46	Aromadendrene	1448	1441 [27]	1617	1620 [28]	-	-	0.3	Sesquiterpene	a, b
47	α-Humuleno	1468	1468 [29]	1679	1667 [28]	0.9	0.8	0.7	Sesquiterpene	a, b, c
48	Alloaromadendreno	1470	1468 [27]	1654	1649 [28]	-	-	0.4	Sesquiterpene	a, b
49	γ-Gurjunene	1473	1475 [27]	-	1714 [29]	0.3	-	-	Sesquiterpene	a, b
50	γ-Muurolene	1484	1478 [27]	1698	1690 [28]	0.4	0.8	-	Sesquiterpene	a, b
51	Germacrene D	1494	1481 [28]	1720	1708 [28]	5.8	6.1	4.3	Sesquiterpene	a, b, c
52	β-Selinene	1496	1490 [27]	1702	1717 [28]	-	0.6	-	Sesquiterpene	a, b
53	Pentadecane	1500	1500 [27]	1500	1500 [29]	-	1.0	-	Straight-chain alkane	a, b
54	Bicyclogermacrene	1507	1500 [27]	1741	1735 [28]	1.6	1.6	3.2	Sesquiterpene	a, b
55	Cadin-3,9-diene	1509	1518 [29]	1721	1720 [29]	-	-	1.6	Sesquiterpene	a, b
56	Eugenyl acetate	1517	1521 [27]	2264	2263 [29]	-	0.9	-	Phenylpropanoid	a, b
57	γ-Cadinene	1523	1513 [27]	1763	1763 [28]	0.2	-	-	Sesquiterpene	a, b
58	Myristicin	1525	1519 [29]	2257	2261 [28]	-	-	3.9	Phenylpropanoid	a, b
59	δ-Cadinene	1527	1523 [27]	1762	1756 [28]	0.9	-	-	Sesquiterpene	a, b
60	Elemicin	1548	1557 [27]	2232	2231 [28]	-	0.4	0.9	Phenylpropanoid	a, b
61	*trans*-Nerolidol	1565	1563 [27]	2042	2036 [28]	0.8	1.2	0.4	Oxygenated sesquiterpene	a, b, c
62	Germacrene B	1573	1559 [27]	1839	1819 [29]	0.3	-	-	Sesquiterpene	a, b
63	Germacrene D-4-ol	1589	1575 [27]	2054	2057 [28]	0.3	-	2.0	Oxygenated sesquiterpene	a, b
64	Caryophyllene oxide	1597	1583 [27]	1989	1986 [28]	0.1	1.6	0.8	Oxygenated sesquiterpene	a, b, c
65	Viridiflorol	1607	1592 [27]	2088	2090 [28]	-	-	5.3	Sesquiterpene	a, b
66	Guaiol	1608	1600 [27]	2078	2089 [28]	-	0.2	-	Oxygenated sesquiterpene	a, b, c
67	Ledol	1616	1612 [29]	2034	2039 [28]	-	-	1.3	Oxygenated sesquiterpene	a, b
68	Dil-apiole	1622	1620 [27]	-	-	-	-	0.6	Phenylpropanoid	
69	Humulene epoxide II	1624	1608 [27]	2046	2047 [28]	0.3	-	-	Oxygenated sesquiterpene	a, b
70	Selin-6-en-4α-ol	1630	1636 [27]	-	-	-	-	1.0	Oxygenated sesquiterpene	a, b
71	γ-Eudesmol	1645	1632 [27]	2173	2176 [28]	-	1.9	-	Oxygenated sesquiterpene	a, b
72	*epi*-α-Cadinol	1650	1640 [27]	2171	2170 [28]	-	-	0.5	Oxygenated sesquiterpene	a, b
73	Murola-4-en-10-ol	1658	1653 [29]	2201	2187 [29]	0.2	0.4	-	Oxygenated sesquiterpene	a, b
74	α-Cadinol	1664	1654 [27]	2233	2227 [28]	-	-	0.3	Oxygenated sesquiterpene	a, b
75	α-Eudesmol	1672	1653 [27]	2227	2223 [28]	-	4.7	-	Oxygenated sesquiterpene	a, b
76	β-Bisabolol	1678	1671 [29]	2155	2151 [29]	0.2	-	-	Oxygenated sesquiterpene	a, b
77	Apiole	1688	1678 [27]	2487	2446 [29]	-	-	29.6	Phenylpropanoid	a, b
78	Heptadecane	1699	1700 [27]	1700	1700 [28]	-	0.4	-	Straight-chain alkane	a, b
79	2-*cis*-6-*trans*-Farnesol	1723	1723 [28]	2361	2357 [28]	-	13.1	-	Oxygenated sesquiterpene	a, b
80	2-*cis*-6-*trans*-farnesyl acetate	1833	1821 [27]	-	-	-	0.3	-	Oxygenated sesquiterpene	a, b

a Tentative identification based on linear retention indices measured using DB5 (nonpolar) and DB-WAX (polar) columns [21,27,28,29]. b Tentative identification based on mass spectra (MS; electron ionization, 70 eV, >95% coincidence), study of fragmentation patterns, and comparison with MS spectra from [27,28,29] spectral databases. c Confirmatory identification based on standard substances by comparison of their mass spectra and retention times (Tr) with those of the EO components.

### 2.2. Antimicrobial Activity Against M. roreri and P. palmivora

The antimicrobial activity of the three essential oils from *P. glabratum*, *P. friedrichsthalii*, and *P. cumanense* shows promising potential in inhibiting the growth of *M. roreri* and *P. palmivora*. These results are promising due to their impact on cacao production in Colombia and their implications for household economies. The essential oil of *P. glabratum* demonstrated promising antifungal activity against *M. roreri*, with results comparable to those obtained with *P. friedrichsthalii*. The activity of the treatments was directly proportional to their concentration, with the first treatment, T1 (56%), and T2 (72%), achieving inhibition rates above 50%. This is a positive indication that the fungus’s growth can be inhibited at low concentrations. Likewise, T3, T4, and T5 showed inhibition percentages of 86%, 94.67%, and 100%, respectively. The essential oil of *P. friedrichsthalii* showed a high inhibition percentage of 69.26% against fungal growth using the lowest concentration, corresponding to treatment 1 (T1 = 18.7 μg/mL). Treatments T2 (31.3 μg/mL) and T3 (114.7 μg/mL) showed inhibition percentages of 80.11% and 90.78%, respectively, and the treatments using higher concentrations, such as T4 (205.5 μg/mL) and T5 (639.9 μg/mL), resulted in 95.84% and 100% inhibition, respectively. On the other hand, the activity against *P. palmivora* varied significantly. Treatments T1 and T2 showed an inhibition percentage close to 24%; however, T3 showed improved activity, reaching 39.78%, which is an acceptable percentage. For T4, the activity increased, reaching 89.44%, and T5 reached 100%. The results for *P. cumanense* show a somewhat different behavior compared to *M. roreri*, as T1 showed 24% inhibition, while treatment T2 showed 64% inhibition. In contrast, treatments T3, T4, and T5 showed 100% inhibition, highlighting that moderate concentrations such as T3 result in a high percentage of inhibition. However, in contrast, the behavior against *P. palmivora* was similar to that of *P. friedrichsthalii*, with T1 and T2 showing inhibition percentages of 10% and 16%, respectively. Nevertheless, with an increase in T3 concentration, a marked increase to 76% was observed, making this a promising treatment for controlling this microorganism. Finally, the highest concentrations, corresponding to T4 and T5, showed inhibition percentages of 98% and 100%, respectively (Table S1; Supplementary Materials).

These results on the inhibition of the growth of the fungus (*M. roreri*) are promising for the development of fungistatic and fungicidal products, depending on the treatment tested. All three essential oils show promising results starting from T1, except for *P. cumanense*, which showed inhibition percentages of 24%, in contrast to *P. glabratum* and *P. friedrichsthalii*, which exhibited values above 50%. From T3 onwards, all species exhibit high inhibition percentages above 80%, results comparable to those obtained with the commercial product (copper oxychloride).

Each treatment was performed in triplicate, and an average value was determined. The Kolmogorov–Smirnov (K-S) test was used to assess normality using the data obtained. In the cases analyzed regarding the growth diameter of *M. roreri* and *P. palmivora* under the different treatments, a condition of normality was observed (K-S test *p*-value > 0.05), with *p*-values greater than 0.05 [30]. An exception was observed for treatments T5 and T7 (control (+)), which showed *p*-values of 0.000; however, this is acceptable due to the microorganism’s inhibition behavior.

**Figure 2:**
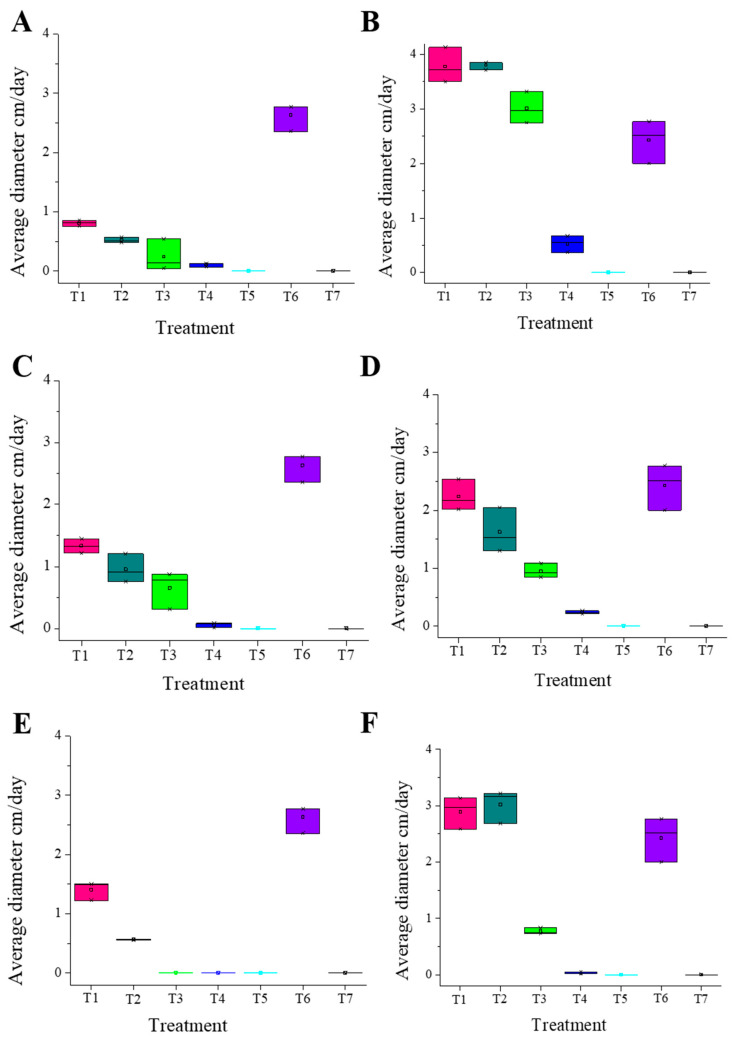
Comparative boxplot of biometric growth on day seven between the five treatments (T1–T5) with essential oil, control (−) T6, and control (+) T7 against *M. roreri* and *P. palmivora*; (**A**) *P. glabratum* essential oil against *M. roreri*; (**B**) *P. glabratum* essential oil against *P. palmivora*; (**C**) *P. friedrichsthalii* essential oil against *M. roreri*; (**D**) *P. friedrichsthalii* essential oil against *P. palmivora*; (**E**) *P. cumanense* essential oil against *M. roreri*; (**F**) *P. cumanense* essential oil against *P. palmivora*.

The experiments for each microorganism were conducted using five treatments and two controls. Each treatment was performed in triplicate, and the average value was calculated for each. The Kolmogorov–Smirnov (K-S) test was applied to the obtained data to verify the normality of the distribution. In the cases analyzed regarding the growth diameter of the fungi *M. roreri* and *P. palmivora* under different treatments, normality was confirmed (*p*-value (K-S test) > 0.05), with *p*-values above 0.05. Treatments T5 and T7 (control (+)) were not considered because they showed *p*-values of 0.000, which is accepted due to the microorganism’s inhibition behavior at the highest concentration of essential oils treatment and commercial antimicrobial (Figure 2) (Table S2; Supplementary Materials).

To confirm the previously obtained values, an analysis of variance (ANOVA) test was first conducted (Table S3; Supplementary Materials), with significance values less than 0.05 and showed highly significant differences across all treatments (*p* < 0.001). The magnitude of these effects was confirmed by Eta squared (η^2^) values ranging from 0.962 to 0.991 for both pathogens (*M. roreri* and *P. palmivora*). These coefficients indicate that the essential oils account for over 96% of the observed variance, representing a large effect size, indicating a significant difference between the means of the diameters in at least two treatments. Additionally, multiple comparison tests (T2—Tamhane) were performed (Tables S5–S10; Supplementary Materials), with results obtained from pairwise comparisons based on the *t*-test to determine the most effective treatment. This was done following a prior verification of non-homogeneity of variances (*p* < 0.05) [30]. This allowed us to calculate effect sizes (η^2^) to quantify the magnitude of treatment effects, while 95% confidence intervals were determined for mean inhibition values. These complementary metrics provide a clearer assessment of the biological relevance of the differences observed between treatments, facilitating comparison beyond statistical significance and reinforcing the robustness and clarity of the data analysis. Therefore, it is concluded that the treatments showing promising inhibitory behavior are those with significant differences (*p* < 0.05) found between T3 and T5 compared to the other treatments. However, it is worth noting that T3 and T4 are promising for the development of products aimed at controlling the proliferation of *M. roreri*, unlike what is observed with *P. palmivora*, where T4 and T5 concentrations show the highest level of microorganism growth control. It should be noted that treatments T5 and T7 were excluded from post hoc statistical comparisons because both produced complete growth inhibition (100%) in all pathogens evaluated, resulting in zero variance and *p*-values of 0.000. Under these conditions, inclusion in multiple comparison analyses is not statistically significant and may distort the interpretation of treatment-dependent differences. From a biological standpoint, the extreme inhibition observed for T5 is due to the high concentration tested, which exceeded the tolerance threshold of all microorganisms and induced a fungicidal response. Similarly, T7 corresponds to the positive control (commercial fungicides), whose established modes of action are expected to cause total inhibition under in vitro conditions. The exclusion of these treatments allows for clearer discrimination between intermediate concentrations, where dose–response relationships and specific pathogen sensitivities can be reliably assessed.

The abundance of chemical compounds with biological activity, particularly antimicrobial activity, has been reported in species of the genus *Piper* and is well-documented in the scientific literature [31]. Numerous studies have investigated the essential oils of *Piper* species, which exhibit a range of chemical cores. The three species evaluated in this study are *P. glabratum*, *P. friedrichsthalii*, and *P. cumanense*, which have distinct chemical profiles but share several biologically important compounds. *P. glabratum* is a species with some previous studies on its essential oils, particularly in relation to its anti-inflammatory and toxic activity [32]. In one study of a specimen collected in Brazil, 61 compounds were identified, with the main components being β-pinene, β-damascenone, *trans*-β-caryophyllene, β-copaene, viridiflorene, and longiborneol. Among these, β-pinene and longiborneol were the predominant compounds.

**Figure 3:**
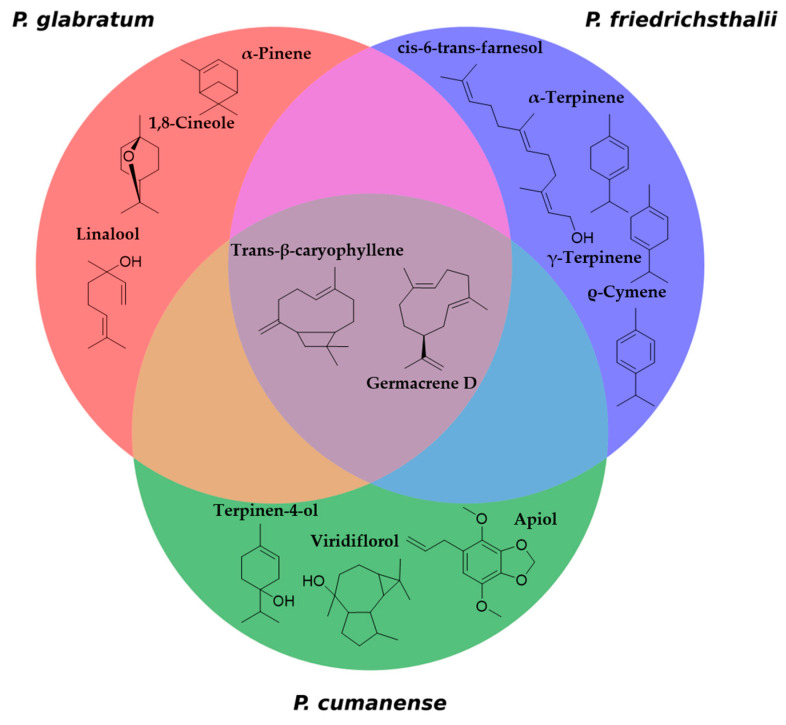
Venn diagram of major compounds, identifying similar and different active ingredients in the three *Piper* species.

In the present study of species collected in the department of Tolima, Colombia, the chromatographic profile shows significant differences in the major components, highlighting that the agroecological conditions of each location have not been considered. This results in variations in the composition and abundance of the primary metabolites present in each essential oil. In this case, the predominant active principles are α-pinene, 1,8-cineole, linalool, *trans*-β-caryophyllene, and germacrene D. *P. friedrichsthalii* has previously studied the composition of the essential oil from individuals collected in Panama, where 35 compounds were identified, and in Costa Rica, where 45 compounds were identified [33]. These studies showed differences in the composition from each location; however, germacrene D is present in the oil of both collections with considerable abundance. The specimen collected in Colombia exhibited a diverse range of chemical components in its essential oil, comprising a total of 44 compounds, of which 41 were successfully characterized. The most abundant compounds were γ-terpinene, α-terpinene, terpinolene, ρ-cymene, germacrene D, *trans*-β-caryophyllene, and *cis*-6-*trans*-farnesol. In the case of *P. cumanense*, studies on essential oil characterization are more limited and focus on identifying compounds derived from benzoic acid [34]. In this case, 47 compounds were identified, with the most representative being terpinen-4-ol, *trans*-β-caryophyllene, germacrene D, viridiflorol, and apiol (Figure 3).

Notably, the chemical richness observed, along with its relationship to antimicrobial activity, suggests that compounds such as γ-terpinene, α-terpinene, terpinolene, *p*-cymene, α-pinene, and β-pinene inhibit the oxygen consumption of microorganisms. This activity is attributed to their synergistic interactions, which effectively inhibit the growth of phytopathogenic fungi and bacteria [35]. It should be noted that the main compounds in the essential oil are 1,8-cineole and α-pinene, mainly present in *P. glabratum*. 1,8-cineole has been shown to be effective in inhibiting phytopathogenic fungi of the genus *Fusarium* and *Aspergillus* [36,37]. It has been shown to permeate the cell membrane and damage organelles without affecting membrane potential. In addition, it has shown antibiofilm activity in *Candida* species, the mechanism of which includes ROS generation and cell cycle arrest [38]. Alternatively, α-pinene has demonstrated fungicidal activity against *Candida albicans*. The mechanism of action is hypothetically associated with ergosterol complexation, a mechanism related to biofilm inhibition through gene downregulation [39]. This demonstrates that 1,8-cineole and α-pinene have antifungal activity using shared inhibition pathways or acting synergistically. Compound such as γ-terpinene, which have already demonstrated antifungal, antibacterial, and antioxidant activities [40].

The antifungal activity of *P. friedrichsthalii* may be attributable to α-terpinene and γ-terpinene, which are the main compounds in the essential oil of this species. Especially γ-Terpinene has antifungal properties demonstrated in laboratory studies on fungi such as *Candida albicans* and *Aspergillus niger*, its mechanism focusing on affecting the cell membrane [41]. The species *P. cumanese* in this study is characterized by having apiol as the main component of its essential oil, a compound known for its toxicity [42]. However, it is a potent antifungal compound due to its ability to alter key metabolic and structural processes in fungi. It mainly inhibits ergosterol biosynthesis by binding to the enzyme lanosterol-14-α-demethylase, causing loss of membrane integrity and deterioration of cell function. In addition, it reduces levels of methylglyoxal, a molecule that triggers oxidative stress responses and aflatoxin production, thereby weakening fungal metabolism. Its molecular interactions with enzymes such as polyketide synthase and Ver-1 suppress aflatoxin biosynthesis. Another interesting compound found in *P. glabratum* and *P. friedrichsthalii* is β-pinene, a compound that has proven effective in reducing conidia production [43,44]. Other compounds of the sesquiterpenes group, such as germacrene D and *trans*-β-caryophyllene [22,45,46], are present in the oils of the three species. Additionally, it has been demonstrated that germacrene D exhibits antimicrobial activity, an assertion supported by multiple published studies in which the tested essential oils are rich in this compound, its mechanism of action is mainly associated with its high lipophilic affinity, which allows it to destabilize the phospholipid bilayer of microbial membranes, inducing ion leakage, membrane depolarization, and metabolic failure that lead to cell death [46,47].

**Figure 4:**
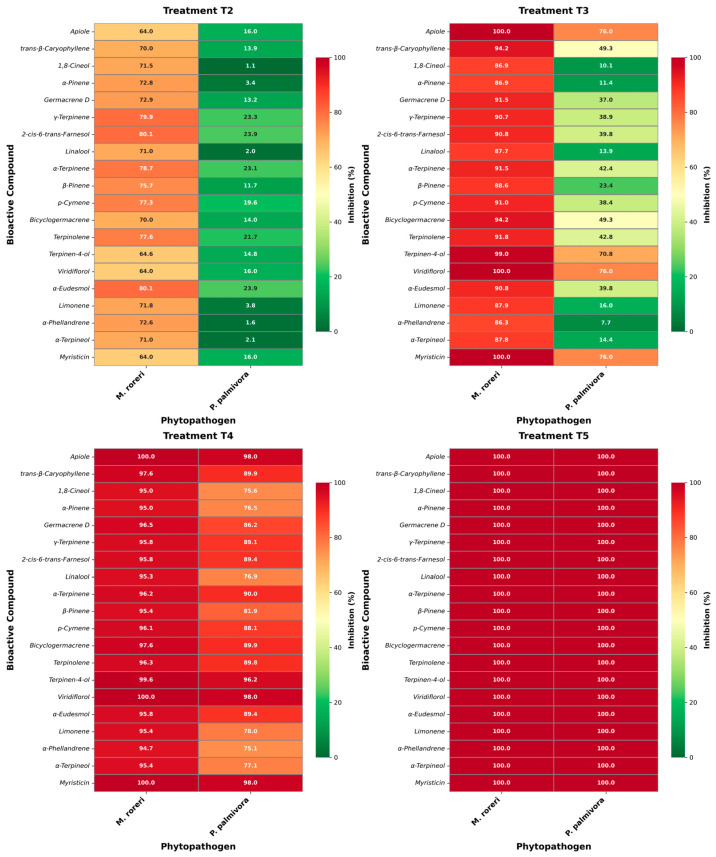
Heatmaps of the structure-activity relationship vs. inhibition percentage of major compounds in essential oils of *Piper* species.

By delving deeper into the analysis, we generate a heat map that represents the relationship between inhibition percentages and the main compounds. This approach allows us to integrate both the relative abundance of each compound and its corresponding level of inhibition [48]. The structure-activity relationship analysis based on the heatmap reveals a clear improvement in concentration-dependent antifungal efficacy in the four treatments (T2–T5) where greater effectiveness is shown, with notable differences between the fungal species *M. roreri* and the oomycete *P. palmivora*. In T2, inhibition against *M. roreri* is already moderate to high for most monoterpenes and sesquiterpenes (≈65–80%), while activity against *P. palmivora* remains low (<25%), highlighting its greater intrinsic tolerance to sublethal concentrations. Compounds such as γ-terpinene, α-terpinene, and 2-*cis*-6-*trans*-farnesol show comparatively better performance, suggesting that increased lipophilicity and unsaturation favor early interactions with the membrane. T3 marks a turning point, at which the percentage of inhibition of *M. roreri* approaches or reaches 100% for multiple compounds (apiol, viridiflorol, myristicin), while *P. palmivora* shows heterogeneous responses. It should be noted that oxygenated terpenoids (terpinen-4-ol, apiol, viridiflorol) show substantially higher activity against *P. palmivora* than hydrocarbon monoterpenes, indicating the importance of polar functionalities in overcoming oomycete defenses. In T4, inhibition becomes uniformly high (>90%) for both phytopathogens, reflecting synergistic effects between structurally related compounds, particularly sesquiterpenes and phenylpropanoids, while for T5, the percentage of inhibition is 100% for all compounds and phytopathogens, concluding that shared physicochemical properties (high lipophilicity, low TPSA, and conserved cyclic structures) dominate activity rather than individual molecular differences. This pattern supports a membrane-driven multi-target mode of action with synergistic reinforcement at higher doses (Figure 4).

To contextualize the relevance of these findings for sustainable cocoa disease management, it is important to consider potential challenges associated with field application. Based on the strong in vitro antifungal activity observed, we recommend further development of the tested formulations as complementary or alternative control tools within integrated disease management programs. However, essential oil–based products may face limitations related to chemical stability and volatility, as rapid evaporation and degradation under field conditions (UV radiation, temperature, rainfall) can reduce persistence and efficacy. Formulation strategies such as microencapsulation, emulsifiable concentrates, or carrier-based delivery systems should therefore be explored to enhance stability and controlled release. Additionally, potential phytotoxic effects must be carefully evaluated, particularly at higher concentrations that showed complete inhibition in vitro, to ensure crop safety under greenhouse and field conditions. Dose optimization and application timing will be critical to balance antifungal efficacy with plant tolerance. Addressing these challenges through formulation improvement and field validation will be essential for translating the promising laboratory results into practical, environmentally friendly solutions for sustainable cocoa disease management.

### 2.3. Cheminformatics Piper Species Essential Oils Analysis

To better understand the activity of essential oils, a study was conducted analyzing the essential oil database of *Piper* species (Table 2, Figure S4; Supplementary Materials), providing valuable information on the molecular characteristics of these natural compounds. The molecular weight (MW) distribution ranges from 100.1 to 264.4 Da, with an average of 181.1 Da. The 80th, 90th, and 95th percentiles (220.7, 222.3, and 222.3 Da, respectively) indicate that most compounds have molecular weights below 220.7 Da, with a relatively small proportion exceeding this value. The topological polar surface area (TPSA), which reflects hydrogen bonding capacity and overall molecular polarity, ranges from 0.0 to 36.9 Å^2^, with an average of 11.5 Å^2^ [49]. The low mean TPSA suggests that most components of these essential oils are relatively nonpolar, which aligns with their known volatility and lipophilic nature. The number of rotatable bonds (NRB) ranges from 0 to 14, with an average of 2, indicating that most compounds possess limited conformational flexibility. This is consistent with the predominance of rigid terpenic structures in essential oils. LogP values, which represent the octanol-water partition coefficient, range from 1.3 to 6.8, with an average of 3.4 [50,51].

This confirms the lipophilic nature of these compounds, which contributes to their ability to penetrate biological membranes. The number of hydrogen bond acceptors (HBA) ranges from 0 to 4, with an average of 1, while the number of hydrogen bond donors (HBD) ranges from 0 to 1. These values reflect the limited hydrogen bonding capacity of most essential oil components, which typically contain few functional groups capable of forming hydrogen bonds. The percentile data for these descriptors reveal that the molecular diversity within the essential oils of the *Piper* species is limited, as most compounds exhibit similar physicochemical properties.

Analysis of the essential oils of *Piper* species reveals significant diversity in molecular properties. Compounds with the minimum molecular weight include *trans*-hex-2-en-1-ol and *cis*-hex-3-en-1-ol (100.16 Da), while 2-*cis*-6-*trans*-farnesyl acetate represents the maximum molecular weight (264.41 Da). As for the topological polar surface area, β-elemene, cadin-3,9-diene, germacrene B, and cyclosativene show minimum values (0.00 Å^2^), while dil-apiole and apiole show maximum values (36.92 Å^2^). Compounds without rotary bonds include camphor, δ-terpineol, isoborneol, and camphene hydrate, while heptadecane possesses the maximum (14.00). LogP values range from 1.33 for *cis*-hex-3-en-1-ol and *trans*-hex-2-en-1-ol to 6.88 for heptadecane, indicating a moderate to high lipophilicity spectrum. The hydrogen bond acceptor capacity varies from zero in compounds such as β-elemene, germacrene D, and δ-cadinene to four in dil-apiole and celery. Similarly, the hydrogen bond donor capacity varies from zero in β-elemene, α-copaene, and methyleugenol to one in compounds such as terpinene-4-ol, borneol, and *cis*-hex-3-en-1-ol. This molecular diversity explains the wide range of biological activities observed in the essential oils of *Piper*, including antimicrobial, antioxidant, and insecticidal properties, making them valuable for various applications ranging from pharmaceuticals to natural pesticides [31].

**Figure 5:**
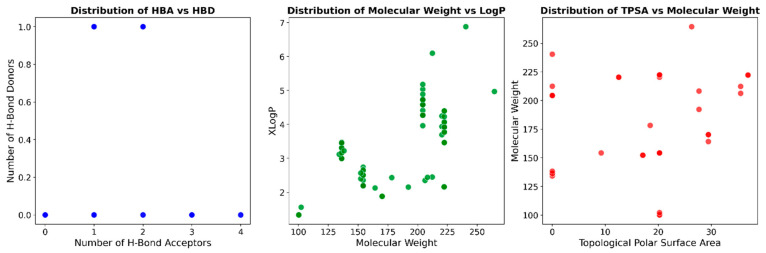
Distribution of *Piper* species essential oils database in terms of the (**left**) number of HBA and HBD, (**center**) MW and LogP, and (**right**) TPSA and MW.

The distribution pattern of essential oils from Piper species, shown in Figure 5, reveals important chemotaxonomic insights. The central graph, which plots molecular weight against LogP, displays four distinct clusters that indicate natural groupings of compounds with similar physicochemical properties. These groups are likely to represent different chemical classes commonly found in *Piper* essential oils, such as monoterpenes, sesquiterpenes, phenylpropanoids, and alkanes/alkenes. This clustering pattern aligns with published research [52,53] which shows that *Piper* essential oils contain diverse classes of compounds, including monoterpene hydrocarbons (α-pinene, β-pinene, limonene), oxygenated monoterpenoids (1,8-cineole, linalool), sesquiterpene hydrocarbons (β-caryophyllene, α-humulene, germacrene D), oxygenated sesquiterpenoids (caryophyllene oxide, nerolidol), and phenylpropanoids (safrole, dillapiole, eugenol, methyl eugenol). The other graphs in Figure 3, which show the distribution of HBA versus HBD and TPSA versus MW, provide complementary information on hydrogen bonding capacity and polarity of these compounds.

**Figure 6:**
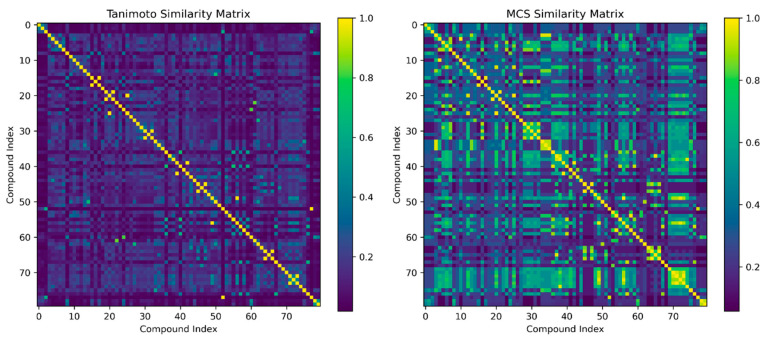
Visualization of Tanimoto and MCS similarity matrices highlights molecular connections within the essential oil database of *Piper* species through structured heatmaps.

The analysis of molecular similarities (Figure 6) in the essential oils of *Piper* species reveals critical structural relationships through Tanimoto and Maximum Common Substructure (MCS) metrics. The Tanimoto similarity matrix identifies compounds with identical molecular fingerprints (Tanimoto = 1.0), including stereoisomers such as *cis*-β-ocimene and *trans*-β-ocimene, as well as structural analogs like iso-borneol/borneol. High MCS scores (≥0.93) highlight shared core scaffolds, especially between pentadecane/heptadecane (MCS 0.94) and γ-cadinene/murola-4-en-10-ol (MCS 0.97), reflecting conserved hydrocarbon structures. Moderately similar pairs, such as eugenol/methyl eugenol (Tanimoto 0.70, MCS 0.96), demonstrate functional group modifications that influence their bioactivity profiles, while lower-scoring pairs, like β-phellandrene/germacrene D (Tanimoto 0.68), reveal divergent terpene architectures. Notably, Tanimoto scores of 1.0 for epi-α-cadinol/α-cadinol and viridiflorol/ledol suggest that these diastereomers may play interchangeable roles in antimicrobial interactions. The MCS analysis further uncovers unexpected pharmacophore overlaps, such as fenchone/camphor (MCS 0.76), which share bicyclic motifs, and eugenol derivatives that show partial alignment with sesquiterpene alcohols (MCS 0.64). These similarity patterns suggest potential synergistic effects among structurally related compounds, particularly within the phenylpropanoids (eugenol/methyl eugenol/eugenyl acetate cluster) and sesquiterpenes (germacrene D and caryophyllene derivatives). The prevalence of highly similar alkane pairs (pentadecane/heptadecane) underscores their role as conserved structural backbones that influence membrane permeability. These molecular relationships provide a foundation for the rational formulation of *Piper*-based biocontrol agents, where similarity clusters could guide the selection of compound combinations with optimized bioavailability and specificity against cacao pathogens such as *M. roreri*.

**Figure 7:**
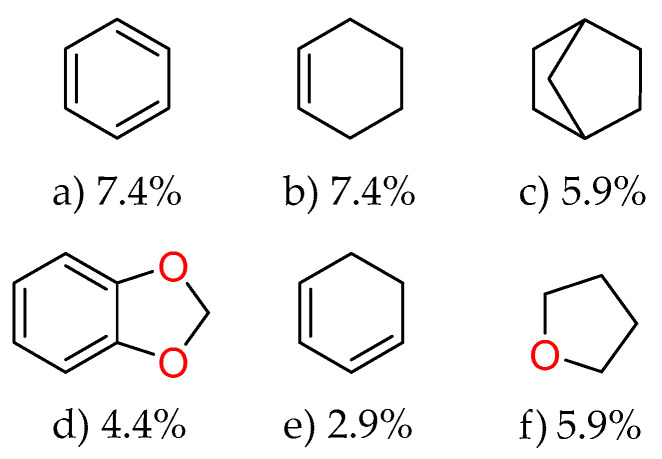
Predominant ring-containing scaffolds identified in the *Piper* species essential oils database. The frequency of each scaffold (as a percentage) is shown below its chemical structure.

The analysis of predominant ring-containing scaffolds (Figure 7) in the essential oils of *Piper* species reveals fascinating insights into their chemical composition. Benzene and cyclohexene emerge as the most prevalent structures, each accounting for 7.4% of the identified compounds. This high frequency of benzene-based structures aligns with the well-known abundance of aromatic compounds in essential oils, particularly phenylpropanoids and benzenoids. The significant presence of cyclohexene scaffolds indicates a substantial proportion of cyclic monoterpenes, which are common in essential oils and often contribute to their characteristic fragrances and biological activities. The bicyclo [2.2.1] heptane scaffold, representing 5.9% of the compounds, suggests the presence of bicyclic terpenes such as camphene or borneol derivatives. The 1,3-benzodioxole scaffold (4.4%) is particularly noteworthy, as it is characteristic of compounds like safrole and its derivatives, which are known bioactive components in many *Piper* species. The cyclohexa-1,3-diene and oxolane scaffolds, each at 2.9%, further diversify the structural landscape, potentially contributing to the complex bioactivities observed in *Piper* essential oils.

**Figure 8:**
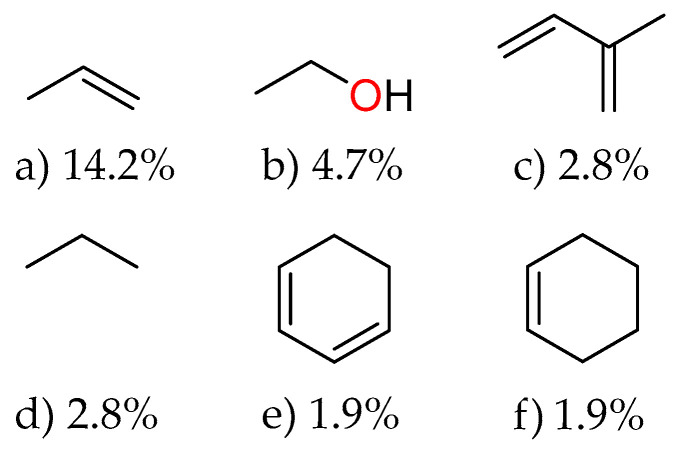
Main fragments extracted from the essential oils database of *Piper* species. Numbers below each structure indicate the relative abundance (as a percentage) of each fragment within the collection.

The analysis of Figure 8 reveals the main fragments present in the essential oils of *Piper* species, providing valuable insights into their molecular composition. The most abundant fragment is prop-1-ene, accounting for 14.2% of the database, indicating the prevalence of allyl groups in these essential oils. This high frequency aligns with the familiar presence of allyl-containing compounds, such as eugenol and safrole, and their derivatives in *Piper* species. Ethanol fragments appear as the second most common, at 4.7%, suggesting the presence of hydroxyl-containing compounds such as alcohols and phenols, which often contribute to the biological activities of essential oils. Fragments of 2-methylbuta-1,3-diene (isoprene) and propane each represent 2.8% of the database, reflecting the terpenic nature of many essential oil components, as terpenes are biosynthetically derived from isoprene units. Cyclohexa-1,3-diene and cyclohexene fragments, each at 1.9%, further confirm the abundance of cyclic monoterpenes in *Piper* essential oils. These cyclohexene-based structures are fundamental building blocks in compounds such as limonene, terpinolene, and phellandrene. The fragment distribution pattern provides a chemoinformatic fingerprint of *Piper* essential oils, highlighting the predominance of olefinic, alcoholic, and cyclic structures.

**Figure 9:**
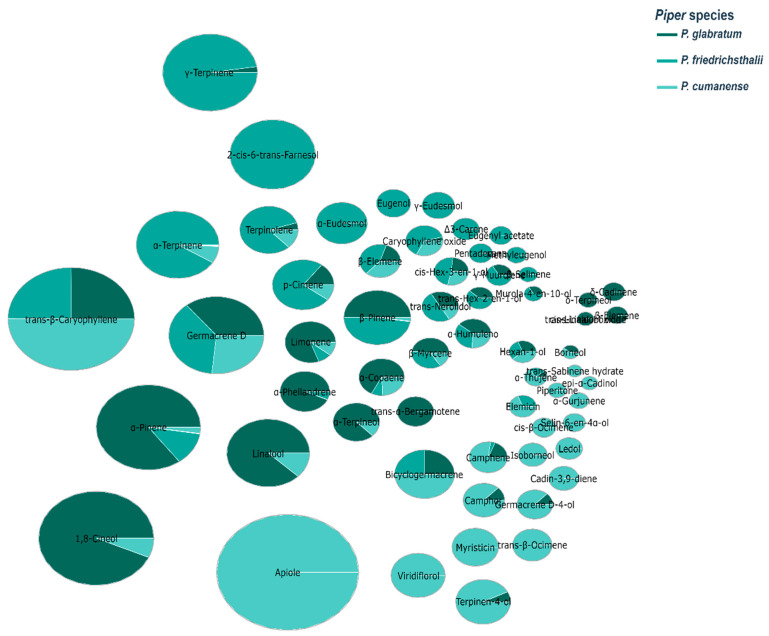
The bubble pie chart illustrates essential oil abundance in *P. glabratum*, *P. friedrichsthalii*, and *P. cumanense*, highlighting interspecies chemical diversity.

Bubble plot analysis (Figure 9) of essential oils from three *Piper* species reveals distinct chemotaxonomic profiles with significant implications for antimicrobial applications against cocoa pathogens. Celery dominates in *P. cumanense* (36.30%), while it is absent in the other species, suggesting species-specific biosynthetic pathways. *trans*-β-Caryophyllene is consistently present in all three species, with the highest concentration in *P. cumanense* (14.40%), indicating its evolutionary conservation within the genus. *P. glabratum* shows a distinctive profile dominated by 1,8-cineole (22.20%) and α-pinene (16.60%), compounds known for their antimicrobial properties against fungi such as *M. roreri*. *P. friedrichsthalii* is characterized by high concentrations of γ-terpinene (15.70%), α-terpinene (11.20%), and the unique presence of 2-*cis*-6-*trans*-farnesol (13.10%). Germacrene D appears consistently in all species (4.30–6.10%), suggesting its fundamental role in the defense mechanisms of the genus *Piper*. The observed chemical diversity correlates with the different antimicrobial activities against cacao pathogens reported in this study, particularly the high efficacy of *P. glabratum* against *M. roreri*. These chemotaxonomic patterns offer valuable insights for the development of species-specific bioproducts to control cacao diseases, potentially replacing conventional fungicides such as copper oxychloride and metalaxyl. The specific chemical signatures of each species could guide selective breeding or extraction protocols to maximize bioactive compounds for the sustainable management of devastating cacao diseases such as moniliasis and black pod rot.

Chemoinformatic results provide a critical mechanistic link between the identified chemical composition and the observed biological activity. The calculated lipophilic profile (average LogP ≈ 3.4) and low topological polar surface area (TPSA ≈ 11.5 Å^2^) indicate that these metabolites possess optimal physicochemical properties for passive diffusion through the fungal cell wall and ergosterol-rich plasma membranes. This high membrane permeability facilitates the accumulation of cytotoxic terpenes within the cytoplasm, leading to the disruption of ionic gradients and cellular leakage. Furthermore, the identification of conserved 1,3-benzodioxole scaffolds (e.g., in apiol and safrole derivatives) is biologically significant; these moieties are known to act as inhibitors of cytochrome P450-dependent detoxification enzymes. This suggests a synergistic mode of action where phenylpropanoids suppress the pathogen’s metabolic resistance, thereby enhancing the susceptibility of *M. roreri* and *P. palmivora* to the membrane-destabilizing effects of the co-occurring monoterpenes [54,55]. To conclusively demonstrate synergistic activity, future studies should explicitly test this hypothesis using factorial bioassays, isobolograms, and combination index models with purified compounds. These approaches would allow for quantitative validation of synergistic, additive, or antagonistic interactions and would reinforce the mechanistic understanding of essential oil-based antifungal activity.

This work is aligned with the public policies proposed by the Colombian government since 2019, focusing on bioeconomy as a development axis, bioprospecting studies should be increased, and promising species like those in this study should be used as a basis for bioeconomic development [56].

## 3. Materials and Methods

### 3.1. Plant Material

The species *P. glabratum* was collected in the district of Buenos Aires, municipality of Ibagué, Colombia (4°18′26.46″ N, 75°6′10.21″ W), in January 2022. The species was collected by Andrea Jiménez-González, Marcial Fuentes-Estrada, and Olimpo García-Beltrán. Botanist Héctor Esquivel carried out the identification, and a specimen is deposited at the TOLI Herbarium of the Universidad del Tolima (Colombia) under voucher number 32,167. *P. friedrichsthalii* (04°21′50′′ N 75°21′17″ W) was collected in the village of Laureles in the municipality of Ibagué. Laura Lugo, Marcial Fuentes-Estrada, and Olimpo García Beltrán collected the species. Botanist Héctor Esquivel identified the specimen, and a sample with voucher No. 32,161 has been deposited at the TOLI Herbarium of the Universidad del Tolima. *P. cumanense* was collected in the district of Buenos Aires (4°19′21″ N 75°5′12″ W) in the municipality of Ibagué. The species was collected by Paloma Sendoya, Marcial Fuentes Estrada, and Olimpo García Beltrán. Botanist Héctor Esquivel identified the specimen, and a sample with voucher No. 32,166 has been deposited at the TOLI Herbarium of the Universidad del Tolima.

### 3.2. Extraction of Essential Oil

*P. glabratum*, *P. friedrichsthalii,* and *P. cumanense* leaf samples were pre-cleaned. The essential oils were then extracted using a microwave-assisted hydrodistillation (MWHD) system [57]. The heating source for the system was a conventional microwave oven (Samsung model MS23J5133AG, Malaysia), set at 2450 MHz and 1.2 kW. A total of 500 mL of distilled water was added to 800 g of plant material. The extraction was carried out in three stages; the first stage lasted 10 min at 100% wave power, while the second and third stages lasted 15 min at 80% microwave power, with 12 min until the temperature of the chiller reached 8 °C.

### 3.3. Chromatographic Analysis

The extracted *P. glabratum*, *P. friedrichsthalii*, and *P. cumanense* essentials oil (50 mg) was dissolved in 1 mL CH_2_Cl_2_, an aliquot of this dilution (2 µL) was injected into a GC 6890 Plus (Agilent Technologies, AT, Palo Alto, CA, USA), equipped with a mass selective detector MS 5973 Network (AT, Palo Alto, CA, USA), using electron ionization (EI, 70 eV). Helium (99.995%, AP gas, Messer, Bogotá, Colombia) was used as carrier gas, with an initial inlet pressure at the column head of 113.5 kPa; the volumetric flow rate of carrier gas during the chromatographic run was kept constant (1 mL/min). The injection mode was split (30:1), and the injector temperature was maintained at 250 °C.

The separation of compounds was carried out on two capillary columns, one with the polar stationary phase of poly(ethylene glycol), PEG (DB-WAX, J & W Scientific, Folsom, CA, USA) of 60 m × 0.25 mm (i.d.) × 0.25 μm (d_f_) of 60 m × 0.25 mm (i.d.) × 0.25 μm (d_f_) and, the second with the stationary phase apolar 5%-phenyl-poly(methylsiloxane), 5%-Ph-PDMS (DB-5MS, J & W Scientific, Folsom, CA, USA) of the exact dimensions. On the DB-WAX column, the oven temperature was programmed from 50 °C (5 min) to 150 °C (7 min), at 4 °C/min, and then to 230 °C (50 min), at 4 °C/min. On the DB-5MS column, the chromatographic oven temperature was programmed as follows: from 45 °C (5 min) to 150 °C (2 min) at 4 °C/min, then to 300 °C (10 min) at 5 °C/min. The GC/MS transfer line temperature was set at 230 °C when using the polar column and at 300 °C for the non-polar column. The ionization chamber and quadrupole temperatures were 250 °C and 150 °C, respectively. The mass range for the acquisition of the ionic currents was *m/z* 45–450 u, with an acquisition rate of 3.58 scan/s. The data were processed with MSDChemStation G1701DA software (AT, Palo Alto, CA, USA). The integration parameters were as follows: threshold = 18 and a “rejection area” of the peak above the baseline of less than 1%. Compound identification was performed based on their linear retention indices (LRI), calculated from the retention times of the compound of interest and the n-alkanes C6-C25 and C8-C40 (Sigma-Aldrich, St. Louis, MO, USA).

For tentative identification, the experimentally obtained mass spectra of each compound were compared with spectral data libraries. Confirmatory identification of some detected compounds was performed by comparing their linear retention indices and mass spectra with standards.

### 3.4. Antimicrobial Activity

In vitro antimicrobial activity was performed against *Moniliophthora roreri* and *Phytophthora palmivora*, microorganisms that affect cocoa production in Colombia and other cocoa-producing countries.

#### 3.4.1. Isolation of *Moniliophthora roreri* and *Phytophthora palmivora*

To obtain the inoculum of *M. roreri* and *P. palmivora*, it is necessary to collect affected fruits from the field that show early symptoms and signs of infection, such as a dark brown spot accompanied by mycelium on the pod’s surface.

The collected fruits will be washed and disinfected with 1% sodium hypochlorite for 2 min. They will then be rinsed with distilled water and dried with sterile absorbent paper. The disinfected fruits will be sectioned (endodermal tissue) into 5 mm in diameter portions in a laminar flow hood. The tissue segments infested with *M. roreri* will be placed in Petri dishes containing potato dextrose agar (PDA) medium and incubated at 25 °C until fungal growth appears in the samples. Identification will be performed based on macroscopic and microscopic characteristics, and the process will be repeated until pure cultures are obtained.

An isolate of *P. palmivora* from the FEDECACAO collection will be used to obtain the inoculum. For the reactivation, purification, and subculturing of the isolate, healthy cacao leaves will be collected from the field, previously disinfected, inoculated, and placed in a humid chamber until symptoms such as initial spots, necrotic lesions, and yellowing appear. Once the *Phytophthora* isolates are obtained, their purity is ensured by performing single-spore cultures [58].

Petri dishes with carrot agar will be incubated for 48 h at 18 °C. Using a Carl Zeiss microscope (Axio Lab.A1, Suzhou, China) with a 40× objective, a conidium is located and a cut is made with a scalpel to extract a single spore, which is then transferred to a new culture medium for seven days.

#### 3.4.2. Antifungal Activity Against *Moniliophthora roreri* and Antimicrobial Activity Against *Phythophthora palmivora*

The in vitro assay of the antifungal activity of the essentials oils from *P. glabratum*, *P. friedrichsthalii*, and *P. cumanense* against *M. roreri* will be conducted under controlled conditions using the poisoned food technique [59], The concentrations tested in the assay are T1–T7, and each treatment will be performed in triplicate, Once the poisoned medium is prepared, 5 mm diameter mycelial disks, obtained from pure cultures of the pathogen with 7 to 10 days of growth, will be placed at the center on the Petri dished containing the treatments. As a negative control of microbial growth, pathogen disks will be placed on PDA medium without essential oils. The treatment evaluated as a positive control will be copper oxychloride, a commercial fungicide known to be activite against *M. roreri*. The Petri dishes will be incubated at 25 °C. Radial growth (in cm) will be measured daily [26]. Measurement will end when the mycelium completely covers the surface of the negative control plate.

The inhibition of mycelial growth of the pathogen is calculated as the percentage of radial growth compared to the control, using Equation (1).

(1) $$\%\;Inhibition=\frac{\left(NCD-TD\right)}{NCD}\times 100$$

where

*NCD* = Negative control diameter;

*TD* = Treatment diameter.

The measurement will be completed when the pathogen’s mycelium completely covers the plate in the control treatment. The evaluation of the product’s effectiveness will be expressed as the percentage of mycelial growth inhibition.

For the development of this activity with *P. palmivora*, the methodology proposed for *M. roreri* will be followed. Agar-carrot culture medium will be prepared, and the plates will be incubated at 25 °C.

#### 3.4.3. Statistical Analysis

The statistical analysis was conducted using a single-factor experimental design and statistical analysis tools through IBM SPSS version 25.0 software. This analysis was applied to the in vitro biometric measurement data obtained from the application of essential oils from *P. glabratum*, *P. friedrichsthalii*, and *P. cumanense*, using five treatments (in triplicate): T1, T2, T3, T4, and T5. In addition to the five different dosage treatments, two control treatments were used: *M. roreri* in PDA (T6) and a commercial fungicide (copper oxychloride) (T7). For the *P. palmivora* assays, the same treatments (T1–T5) were used, with only the culture medium changed (carrot-agar in T6 and T7, enriched with metalaxyl).

Initially, the Kolmogorov–Smirnov (K-S) test was applied to measurements to verify normality (with a *p*-value (K-S test) > 0.05). Likewise, the Levene’s test was used to check homogeneity of variances in order to determine equality or difference between at least one pair of treatment means, and thus apply one-way ANOVA to test specific differences between treatments using post hoc tests (DMS or Tamhane’s T2), accepting significant differences with a *p*-value < 0.05.

### 3.5. Cheminformatics Details

The study methodology was based on computational analysis using chemoinformatics. The 80 canonical SMILES of essential oils from *Piper* were obtained from PubChem (access: 10 January 2025). Molecular descriptor calculations (MW, TPSA, NRB, LogP, HBA, HBD) were performed using RDKit 2024.9.6 in Python 3.10.14, with pandas 2.2.1 for data handling. Structural similarities were evaluated using Tanimoto indices (threshold ≥ 0.6) based on Morgan fingerprints (radius = 2, 1024 bits) and maximum common substructure (MCS) (requiring full match of rings and valences). Scaffolds were identified using the Murcko algorithm, while molecular fragmentation was limited to 3 non-cyclic single bonds, excluding structures with fewer than three atoms. For visualization, a bubble pie chart was generated using t-SNE (scikit-learn 1.6.1) with a perplexity of 15, optimizing the layout with a force-directed algorithm to minimize overlap (radius proportional to abundance, scale factor = 6). This was implemented using NumPy 1.26.4 and Plotly 6.0.1 for interactive rendering.

## 4. Conclusions

The present study demonstrates that the essential oils of *P. glabratum*, *P. friedrichsthalii*, and *P. cumanense* exhibit significant antifungal and antimicrobial activity against key phytopathogens affecting *Theobroma cacao*, specifically *M. roreri* and *P. palmivora*. The chemical characterization revealed a rich diversity of bioactive compounds, predominantly monoterpenes (α-pinene, 1,8-cineole), sesquiterpenes (*trans*-β-caryophyllene, germacrene D), and phenylpropenes (apiole). In particular, *P. glabratum* and *P. friedrichsthalii* showed over 50% inhibition of *M. roreri* at low concentrations (4.44 and 18.7 mg/mL, respectively), with activity comparable to that of copper oxychloride, while *P. cumanense* demonstrated high efficacy against *P. palmivora*, similar to that of metalaxyl, a commercially used antimicrobial agent. Chemoinformatic analysis confirmed the predominance of lipophilic, low-polarity, and hydrogen-bonding-limited molecules, supporting their bioavailability and membrane permeability, key factors in antifungal efficacy. The molecular similarity and distribution of the scaffolds revealed conserved and synergistic structures, highlighting their potential as rational leads in the development of bioformulations. These findings underscore the potential of *Piper* essential oils as sustainable alternatives to synthetic agrochemicals, contributing to integrated disease management in cocoa cultivation and aligning with national bioeconomy strategies. Further field validation and formulation optimization are recommended to convert these natural products into scalable biocontrol solutions.

## Figures and Tables

**Table 2:** Calculated percentiles and statistical properties of primary descriptors for the *Piper* species essential oils database.

Descriptor	80th Percentile	90th Percentile	95th Percentile	Maximum	Minimum	Average
MW (Da)	220.7	222.3	222.3	264.4	100.1	181.1
TPSA (Å^2^)	20.2	27.6	29.7	36.9	0.0	11.5
NRB ^[a]^	3	4	7	14	0	2
LogP	4.5	4.7	4.9	6.8	1.3	3.4
HBA ^[b]^	1	2	3	4	0	1
HBD ^[c]^	1	1	1	1	0	1

^[a]^ Number of rotatable bonds. ^[b]^ Number of hydrogen bond acceptors. ^[c]^ Number of hydrogen bond donors.

## Data Availability

The authors may share the datasets obtained in this study upon reasonable request, provided that the conditions are appropriate. For this purpose, they may write to the following e-mail addresses: jose.garcia@unibague.edu.co and oyanez@udla.cl.

## Supplementary Materials

The following supporting information can be downloaded at: https://www.mdpi.com/article/10.3390/molecules31020326/s1, Figure S1. Chromatographic profile obtained by GC/MS (full scan) of the essential oil of *P. glabratum*. DB-5MS column (60 m), 1:30 split, MSD (EI, 70 eV); Figure S2. Chromatographic profile obtained by GC/MS (full scan) of the essential oil of *P. friedrichsthalii*. DB-5MS column (60 m), 1:30 split, MSD (EI, 70 eV); Figure S3. Chromatographic profile obtained by GC/MS (full scan) of the essential oil of *P. cumanense*. DB-5MS column (60 m), 1:30 split, MSD (EI, 70 eV); Figure S4. 2D structure of Essential oils of Piper species; Table S1. Treatments and inhibition percentages of the application of essential oils of *P. glabratum*, *P. friedrichsthalii*, and *P. cumanense* against *M. roreri* and *P. palmivora*; Table S2. Descriptive statistics and normality tests (Kolmogorov-Smirnov) for treatments applied to different Piper species against *M. roreri* and *P. palmivora*; Table S3. Anova test of the essential oils of *P. glabratum*, *P. friedrichsthalii* and *P. cumanense* against *M. roreri* and *P. palmivora*; Table S4. Test of homogeneity of variances of the essential oil *P. glabratum*, *P. friedrichsthalii* and *P. cumanense* against *M. roreri* and *P. palmivora*; Table S5. T2-Tamhane test comparisons of *P. glabratum* essential oil versus *M. roreri*; Table S6. T2-Tamhane test multiple comparisons of *P. glabratum* essential oil against Phytophthora palmivora; Table S7. T2-Tamhane test multiple comparisons of the essential oil of *P. friedrichsthalii* versus *M. roreri*; Table S8. T2-Tamhane test multiple comparisons of the essential oil of *P. friedrichsthalii* versus *P. palmivora*; Table S9. T2-Tamhane test multiple comparisons of the essential oil of *P. cumanense* (p) versus *M. roreri*; Table S10. T2-Tamhane test multiple comparisons of the essential oil of *P. cumanense* (P) versus *P. palmivora*.

## References

[1] Rusconi M., Conti A. (2010). *Theobroma cacao* L., the Food of the Gods: A Scientific Approach beyond Myths and Claims. Pharmacol. Res..

[2] Campos-Vega R., Nieto-Figueroa K.H., Oomah B.D. (2018). Cocoa (*Theobroma cacao* L.) Pod Husk: Renewable Source of Bioactive Compounds. Trends Food Sci. Technol..

[3] Herrera-Rocha F., Fernández-Niño M., Cala M.P., Duitama J., Barrios A.F.G. (2023). Omics Approaches to Understand Cocoa Processing and Chocolate Flavor Development: A Review. Food Res. Int..

[4] Bagnulo E., Scavarda C., Bortolini C., Cordero C., Bicchi C., Liberto E. (2023). Cocoa Quality: Chemical Relationship of Cocoa Beans and Liquors in Origin Identitation. Food Res. Int..

[5] Fanning E., Eyres G., Frew R., Kebede B. (2023). Linking Cocoa Quality Attributes to Its Origin Using Geographical Indications. Food Control.

[6] Henderson J.S., Joyce R.A., Hall G.R., Hurst W.J., Mcgovern P.E. (2007). Chemical and Archaeological Evidence for the Earliest Cacao Beverages. Proc. Natl. Acad. Sci. USA.

[7] International Cocoa Organization (ICCO) Statistics—International Cocoa Organization. https://www.icco.org/statistics/.

[8] Barrera-Ramírez J., Prado V., Solheim H. (2019). Life Cycle Assessment and Socioeconomic Evaluation of the Illicit Crop Substitution Policy in Colombia. J. Ind. Ecol..

[9] Arnold A.E., Herre E.A. (2003). Canopy Cover and Leaf Age Affect Colonization by Tropical Fungal Endophytes: Ecological Pattern and Process in *Theobroma cacao* (Malvaceae). Mycologia.

[10] Ploetz R.C. (2007). Cacao Diseases: Important Threats to Chocolate Production Worldwide. Proceedings of the Phytopathology.

[11] Siaw D., Ofosu G., Sarpong D. (2023). Cocoa Production, Farmlands, and the Galamsey: Examining Current and Emerging Trends in the ASM-Agriculture Nexus. J. Rural. Stud..

[12] Villamizar-Gallardo R., Osma J.F., Ortíz-Rodriguez O.O. (2019). Regional Evaluation of Fungal Pathogen Incidence in Colombian Cocoa Crops. Agriculture.

[13] Jaimes Y.Y., Gonzalez C., Rojas J., Cornejo O.E., Mideros M.F., Restrepo S., Cilas C., Furtado E.L. (2016). Geographic Differentiation and Population Genetic Structure of *Moniliophthora roreri* in the Principal Cocoa Production Areas in Colombia. Plant Dis..

[14] Chitiva-Chitiva L.C., Ladino-Vargas C., Cuca-Suárez L.E., Prieto-Rodríguez J.A., Patiño-Ladino O.J. (2021). Antifungal Activity of Chemical Constituents from *Piper pesaresanum* C. DC. and Derivatives against Phytopathogen Fungi of Cocoa. Molecules.

[15] Arena M., Auteri D., Barmaz S., Bellisai G., Brancato A., Brocca D., Bura L., Byers H., Chiusolo A., EFSA (2018). Peer Review of the Pesticide Risk Assessment of the Active Substance Copper Compounds Copper(I), Copper(II) Variants Namely Copper Hydroxide, Copper Oxychloride, Tribasic Copper Sulfate, Copper(I) Oxide, Bordeaux Mixture. EFSA J..

[16] Norgrove L. (2007). Effects of Different Copper Fungicide Application Rates upon Earthworm Activity and Impacts on Cocoa Yield over Four Years. Eur. J. Soil Biol..

[17] Jaimes Suárez Y.Y., Agudelo Castañeda G.A., Báez Daza E.Y., Rengifo Estrada G.A., Rojas Molina J. (2021). Modelo Productivo Para El Cultivo de Cacao (*Theobroma cacao* L.) En El Departamento de Santander.

[18] Mahecha-Jimenez Y.S., Patiño-Ladino O.J., Prieto-Rodríguez J.A. (2025). Chemical Constituents and Antifungal Properties of *Piper ceanothifolium* Kunth Against Phytopathogens Associated with Cocoa Crops. Plants.

[19] Luca S.V., Gaweł-bęben K., Strzępek-gomółka M., Czech K., Trifan A., Zengin G., Korona-Glowniak I., Minceva M., Gertsch J., Skalicka-woźniak K. (2021). Insights into the Phytochemical and Multifunctional Biological Profile of Spices from the Genus *Piper*. Antioxidants.

[20] Raikwar G., Mohan S., Dahiya P. (2025). Chemical Composition, Antibacterial and Antioxidant Activities of *Piper betle* and *Anethum graveolens* Essential Oils against Methicillin-Resistant *Staphylococcus aureus* Clinical Isolates. Braz. J. Microbiol..

[21] Le N.V., Sam L.N., Huong L.T., Ogunwande I.A. (2022). Chemical Compositions of Essential Oils and Antimicrobial Activity of *Piper albispicum* C. DC. from Vietnam. J. Essent. Oil-Bear. Plants.

[22] Tangarife-Castaño V., Correa-Royero J.B., Roa-Linares V.C., Pino-Benitez N., Betancur-Galvis L.A., Durán D.C., Stashenko E.E., Mesa-Arango A.C. (2014). Anti-Dermatophyte, Anti-Fusarium and Cytotoxic Activity of Essential Oils and Plant Extracts of *Piper* Genus. J. Essent. Oil Res..

[23] Sen S., Rengaian G. (2022). A Review on the Ecology, Evolution and Conservation of *Piper* (Piperaceae) in India: Future Directions and Opportunities. Bot. Rev..

[24] Yongye A.B., Waddell J., Medina-Franco J.L. (2012). Molecular Scaffold Analysis of Natural Products Databases in the Public Domain. Chem. Biol. Drug Des..

[25] Wenderski T.A., Stratton C.F., Bauer R.A., Kopp F., Tan D.S. (2015). Principal Component Analysis as a Tool for Library Design: A Case Study Investigating Natural Products, Brand-Name Drugs, Natural Product-like Libraries, and Drug-like Libraries. Methods Mol. Biol..

[26] Fuentes-Estrada M., Jiménez-González A., Duarte D., Saavedra-Barrera R., Areche C., Stashenko E., Pino Benítez N., Bárcenas-Pérez D., Cheel J., García-Beltrán O. (2023). GC/MS Profile and Antifungal Activity of *Zanthoxylum caribaeum* Lam Essential Oil against *Moniliophthora roreri* Cif and Par, a Pathogen That Infects Theobroma Cacao L Crops in the Tropics. Chemosensors.

[27] Adams R.P. (2012). Identification of Essential Oil Components by Gas Chromatography/Mass Spectrometry.

[28] Babushok V.I., Linstrom P.J., Zenkevich I.G. (2011). Retention Indices for Frequently Reported Compounds of Plant Essential Oils. J. Phys. Chem. Ref. Data.

[29] NIST *NIST/EPA/NIH Spectral Library with Search Program*, Version 2.3; National Institute of Standards and Technology: Gaithersburg, MD, USA. https://books.google.es/books?hl=es&lr=&id=9phHDwAAQBAJ&oi=fnd&pg=PR1&dq=NIST+Standard+Reference+Database.+NIST/EPA/NIH+Spectral+Library+with+Search+Program,+Version+2.3%3B+National+Institute+of+Standards+and+Technology:+Gaithersburg,+MD,+USA,+2017&ots=E5CXV-_HUo&sig=t4n6NnfWw3W4UHPX4yhm-dqp1nQ#v=onepage&q&f=false.

[30] Jayakumar V., Ramesh Sundar A., Viswanathan R. (2021). Biocontrol of *Colletotrichum falcatum* with Volatile Metabolites Produced by Endophytic Bacteria and Profiling VOCs by Headspace SPME Coupled with GC–MS. Sugar Tech.

[31] da Silva J.K., da Trindade R., Alves N.S., Figueiredo P.L., Maia J.G.S., Setzer W.N. (2017). Essential Oils from Neotropical *Piper* Species and Their Biological Activities. Int. J. Mol. Sci..

[32] Branquinho L.S., Santos J.A., Cardoso C.A.L., Mota J.d.S., Junior U.L., Kassuya C.A.L., Arena A.C. (2017). Anti-Inflammatory and Toxicological Evaluation of Essential Oil from *Piper glabratum* Leaves. J. Ethnopharmacol..

[33] Vila R., Mundina M., Tomi F., Cicció J.F., Gupta M.P., Iglesias J., Casanova J., Cañigueral S. (2003). Constituents of the Essentials Oils from *Piper friedrichsthalii* C.DC. and *P. pseudolindenii* C.DC. from Central America. Flavour. Fragr. J..

[34] Parra Amin J.E., Cuca L.E., González-Coloma A. (2021). Antifungal and Phytotoxic Activity of Benzoic Acid Derivatives from Inflorescences of *Piper cumanense*. Nat. Prod. Res..

[35] Soulaimani B. (2025). Comprehensive Review of the Combined Antimicrobial Activity of Essential Oil Mixtures and Synergism with Conventional Antimicrobials. Nat. Prod. Commun..

[36] Hoch C.C., Petry J., Griesbaum L., Weiser T., Werner K., Ploch M., Verschoor A., Multhoff G., Bashiri Dezfouli A., Wollenberg B. (2023). 1,8-Cineole (Eucalyptol): A Versatile Phytochemical with Therapeutic Applications across Multiple Diseases. Biomed. Pharmacother..

[37] Morcia C., Malnati M., Terzi V. (2012). In Vitro Antifungal Activity of Terpinen-4-Ol, Eugenol, Carvone, 1,8-Cineole (Eucalyptol) and Thymol against Mycotoxigenic Plant Pathogens. Food Addit. Contam. Part A.

[38] Shahina Z., Al Homsi R., Price J.D.W., Whiteway M., Sultana T., Dahms T.E.S. (2022). Rosemary Essential Oil and Its Components 1,8-Cineole and α-Pinene Induce ROS-Dependent Lethality and ROS-Independent Virulence Inhibition in *Candida albicans*. PLoS ONE.

[39] de Barros D., de Oliveira e Lima L., da Silva L., Cavalcante Fonseca M., Ferreira R.C., Diniz Neto H., da Nóbrega Alves D., da Silva Rocha W.P., Scotti L., de Oliveira Lima E. (2023). α-Pinene: Docking Study, Cytotoxicity, Mechanism of Action, and Anti-Biofilm Effect against Candida Albicans. Antibiotics.

[40] Sousa L.G.V., Castro J., Cavaleiro C., Salgueiro L., Tomás M., Palmeira-Oliveira R., Martinez-Oliveira J., Cerca N. (2022). Synergistic Effects of Carvacrol, α-Terpinene, γ-Terpinene, ρ-Cymene and Linalool against Gardnerella Species. Sci. Rep..

[41] Wiart C., Kathirvalu G., Raju C.S., Nissapatorn V., Rahmatullah M., Paul A.K., Rajagopal M., Sathiya Seelan J.S., Rusdi N.A., Lanting S. (2023). Antibacterial and Antifungal Terpenes from the Medicinal Angiosperms of Asia and the Pacific: Haystacks and Gold Needles. Molecules.

[42] Dosoky N.S., Setzer W.N. (2021). Maternal Reproductive Toxicity of Some Essential Oils and Their Constituents. Int. J. Mol. Sci..

[43] Amri I., Gargouri S., Hamrouni L., Hanana M., Fezzani T., Jamoussi B. (2012). Chemical Composition, Phytotoxic and Antifungal Activities of *Pinus pinea* Essential Oil. J. Pest Sci. (2004).

[44] Molla Yitayeh M., Monie Wassihun A. (2022). Chemical Composition and Antibacterial and Antioxidant Activities of Stem Bark Essential Oil and Extracts of *Solanecio gigas*. Biochem. Res. Int..

[45] Jaramillo-Colorado B.E., Arroyo-Salgado B., Palacio-Herrera F.M. (2025). Antifungal Activity of Four Piper Genus Essential Oils against Postharvest Fungal *Fusarium* spp. Isolated from *Mangifera indica* L. and *Persea americana* Mill. Fruits. Ind. Crop. Prod..

[46] Grande-Tovar C.D., Chaves-Lopez C., Viuda-Martos M., Serio A., Delgado-Ospina J., Perez-Alvarez J.A., Ospina N., la Tora S., Palmieri S., Paparella A. (2016). Sub-Lethal Concentrations of Colombian *Austroeupatorium inulifolium* (H.B.K.) Essential Oil and Its Effect on Fungal Growth and the Production of Enzymes. Ind. Crop. Prod..

[47] Tanapichatsakul C., Khruengsai S., Pripdeevech P. (2020). In Vitro and in Vivo Antifungal Activity of Cuminum Cyminum Essential Oil against *Aspergillus aculeatus* Causing Bunch Rot of Postharvest Grapes. PLoS ONE.

[48] Garnier E., Lavorel S., Ansquer P., Castro H., Cruz P., Dolezal J., Eriksson O., Fortunel C., Freitas H., Golodets C. (2007). Assessing the Effects of Land-Use Change on Plant Traits, Communities and Ecosystem Functioning in Grasslands: A Standardized Methodology and Lessons from an Application to 11 European Sites. Ann. Bot..

[49] Ertl P., Rohde B., Selzer P. (2000). Fast Calculation of Molecular Polar Surface Area as a Sum of Fragment-Based Contributions and Its Application to the Prediction of Drug Transport Properties. J. Med. Chem..

[50] Constantinescu T., Lungu C.N., Lung I. (2019). Lipophilicity as a Central Component of Drug-Like Properties of Chalchones and Flavonoid Derivatives. Molecules.

[51] Anjana R., Yongqiang Z., Sabina M., Rajini R. (2010). Mechanism of Antifungal Activity of Terpenoid Phenols Resembles Calcium Stress and Inhibition of the TOR Pathway. Antimicrob. Agents Chemother..

[52] Al-Sayed E., Gad H.A., El-Kersh D.M. (2021). Characterization of Four Piper Essential Oils (GC/MS and ATR-IR) Coupled to Chemometrics and Their Anti- *Helicobacter pylori* Activity. ACS Omega.

[53] Almeida C.A., Azevedo M.M.B., Chaves F.C.M., Roseo De Oliveira M., Rodrigues I.A., Bizzo H.R., Gama P.E., Alviano D.S., Alviano C.S. (2018). *Piper* Essential Oils Inhibit *Rhizopus oryzae* Growth, Biofilm Formation, and Rhizopuspepsin Activity. Can. J. Infect. Dis. Med. Microbiol..

[54] Bakkali F., Averbeck S., Averbeck D., Idaomar M. (2008). Biological Effects of Essential Oils—A Review. Food Chem. Toxicol..

[55] Atnafu B., Abedeta C., Lemessa F., Mohammed A., Oufensou S., Chala A. (2024). Chemical Composition of Selected Aromatic Plant Essential Oils and Their Antifungal Efficacy against Toxigenic Fungi Associated with Maize (*Zea mays* L). Cogent Food Agric..

[56] Grilli G., Cantillo T., Turner K., Erazo J., Murcia López M.A., Valle Parra J.S., Cardona F.G., Ferrini S. (2024). A Decision Support Procedure for the Bioeconomy Transition: A Colombian Case Study. J. Environ. Manag..

[57] Martínez A., Manrique-Moreno M., Klaiss-Luna M.C., Stashenko E., Zafra G., Ortiz C. (2021). Effect of Essential Oils on Growth Inhibition, Biofilm Formation and Membrane Integrity of *Escherichia coli* and *Staphylococcus aureus*. Antibiotics.

[58] Fisher N.L., Marasas W.F.O., Toussoun T.A. (1983). Taxonomic Importance of Microconidial Chains in *Fusarium* Section Liseola and Effects of Water Potential on Their Formation. Mycologia.

[59] De la Cruz-López N., Cruz-López L., Holguín-Meléndez F., Guillén-Navarro G.K., Huerta-Palacios G. (2022). Volatile Organic Compounds Produced by Cacao Endophytic Bacteria and Their Inhibitory Activity on *Moniliophthora roreri*. Curr. Microbiol..

