# Supplementary material for: Chemical Profiling and Cheminformatic Insights into Piper Essential Oils as Sustainable Antimicrobial Agents Against Pathogens of Cocoa Crops
Source: Molecules. 2026 Jan 17;31(2):326. doi: 10.3390/molecules31020326 (PMC12844135; doi:10.3390/molecules31020326)
Supplement: Supplementary file 1 [file molecules-31-00326-s001.zip › molecules-3992685-supplementary.pdf]

Supplementary Materials

Chemical Profiling and Cheminformatic Insights into *Piper* Essential Oils as Sustainable Antimicrobial Agents Against Pathogens of Cocoa Crops

Diannefair Duarte[1], Marcial Fuentes-Estrada[2], Yorladys Martínez Aroca[3], Paloma Sendoya-Gutiérrez[3], Manuel I. Osorio[4,5], Osvaldo Yañez[6], Carlos Areche[7], Elena Stashenko[8], and Olimpo García-Beltrán[9,10,*]

1 Facultad de Ciencias Agrarias, Universidad de Pamplona, Carretera a Bucaramanga 1 Km, Pamplona 543050, Colombia

2 Institución Educativa Otoniel Guzmán, Secretaría de Educación del Tolima, Venadillo 730580, Colombia

3 Facultad de Ciencias, Ingeniería e Innovación, Universidad de Ibagué, Carrera 22 Calle 67, Ibagué 730002, Colombia

4 Facultad de Odontología, Universidad Andres Bello, Santiago 8370133, Chile

5 Facultad de Medicina, Universidad Diego Portales, Santiago 8370007, Chile

6 Centro de Modelación Ambiental y Dinámica de Sistemas (CEMADIS), Facultad de Ingeniería y Negocios, Universidad de Las Américas, Santiago 7500975, Chile

7 Departamento de Química, Facultad de Ciencias, Universidad de Chile, Casilla 653, Santiago 3521000, Chile

8 Research Center of Excellence CENIVAM, CIBIMOL, Universidad Industrial de Santander, Building 45, UIS, Carrera 27, Calle 9, Bucaramanga 680002, Colombia

9 Centro de Estudios e Investigación en Salud y Sociedad (EISS), Universidad Bernardo O'Higgins, General Gana 1702, Santiago 8370854, Chile.

10 Colaboratorio de Investigación en Bioeconomía Regional, Universidad de Ibagué, Carrera 22 Calle 67, Ibagué 730002, Colombia

* Correspondence: jose.garcia@unibague.edu.co; +57-8276-0010 (O.G.-B.).

Table of contents

Contents	Page
**Figure S1**. Chromatographic profile obtained by GC/MS (full scan) of the essential oil of *P. glabratum*. DB-5MS column (60 m), 1:30 split, MSD (EI, 70 eV).	3
**Figure S2**. Chromatographic profile obtained by GC/MS (full scan) of the essential oil of *P. friedrichsthalii*. DB-5MS column (60 m), 1:30 split, MSD (EI, 70 eV).	3
**Figure S3**. Chromatographic profile obtained by GC/MS (full scan) of the essential oil of *P. cumanense*. DB-5MS column (60 m), 1:30 split, MSD (EI, 70 eV).	4
**Table S1**. Treatments and inhibition percentages of the application of essential oils of *P. glabratum*, *P. friedrichsthalii*, and *P. cumanense* against *M. roreri* and *P. palmivora*.	4
**Table S2**. Descriptive statistics and normality tests (Kolmogorov-Smirnov) for treatments applied to different *Piper* species against *M. roreri* and *P. palmivora*.	5
**Table S3**. Anova test of the essential oils of *P. glabratum*, *P. friedrichsthalii* and *P. cumanense* against *M. roreri* and *P. palmivora*.	6
**Table S4.** Test of homogeneity of variances of the essential oil *P. glabratum*, *P. friedrichsthalii* and *P. cumanense* against *M. roreri* and *P. palmivora.*	7
**Table S5.** T2-Tamhane test comparisons of *P. glabratum* essential oil versus *M. roreri*.	8
**Table S6.** T2-Tamhane test multiple comparisons of *P. glabratum* essential oil against *Phytophthora palmivora*.	9
**Table S7**. T2-Tamhane test multiple comparisons of the essential oil of *P. friedrichsthalii* versus *M. roreri*.	10
**Table S8.** T2-Tamhane test multiple comparisons of the essential oil of *P. friedrichsthalii* versus *P. palmivora.*	11
**Table S9.** T2-Tamhane test multiple comparisons of the essential oil of *P. cumanense* (p) versus *M. roreri*	12
**Table S10.** T2-Tamhane test multiple comparisons of the essential oil of *P. cumanense* (P) versus *P. palmivora*	13
**Figure S4**. 2D structure of Essential oils of *Piper* specie	17

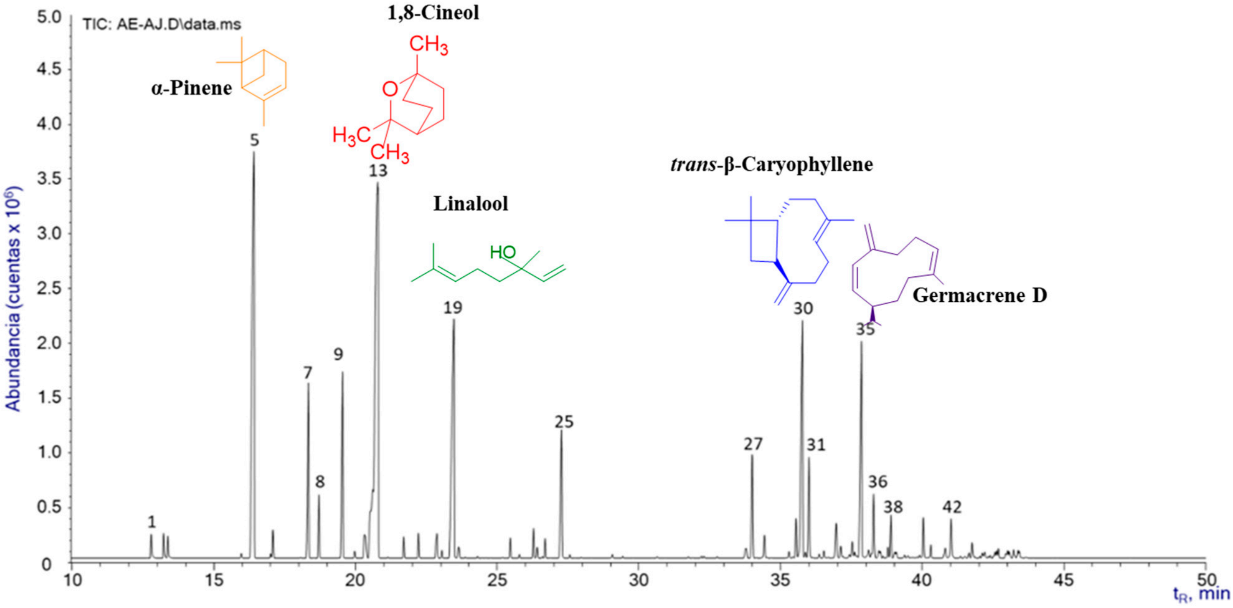

Figure S1. Chromatographic profile obtained by GC/MS (full scan) of the essential oil of *P. glabratum*. DB-5MS column (60 m), 1:30 split, MSD (EI, 70 eV).

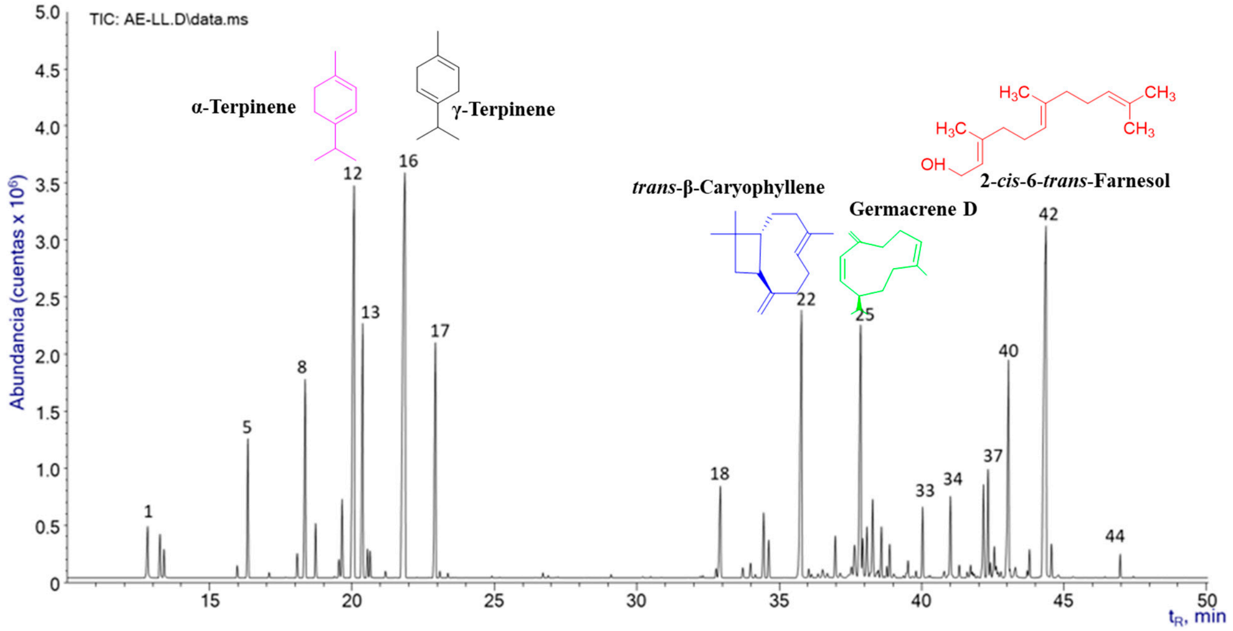

Figure S2. Chromatographic profile obtained by GC/MS (full scan) of the essential oil of *P. friedrichsthalii*. DB-5MS column (60 m), 1:30 split, MSD (EI, 70 eV).

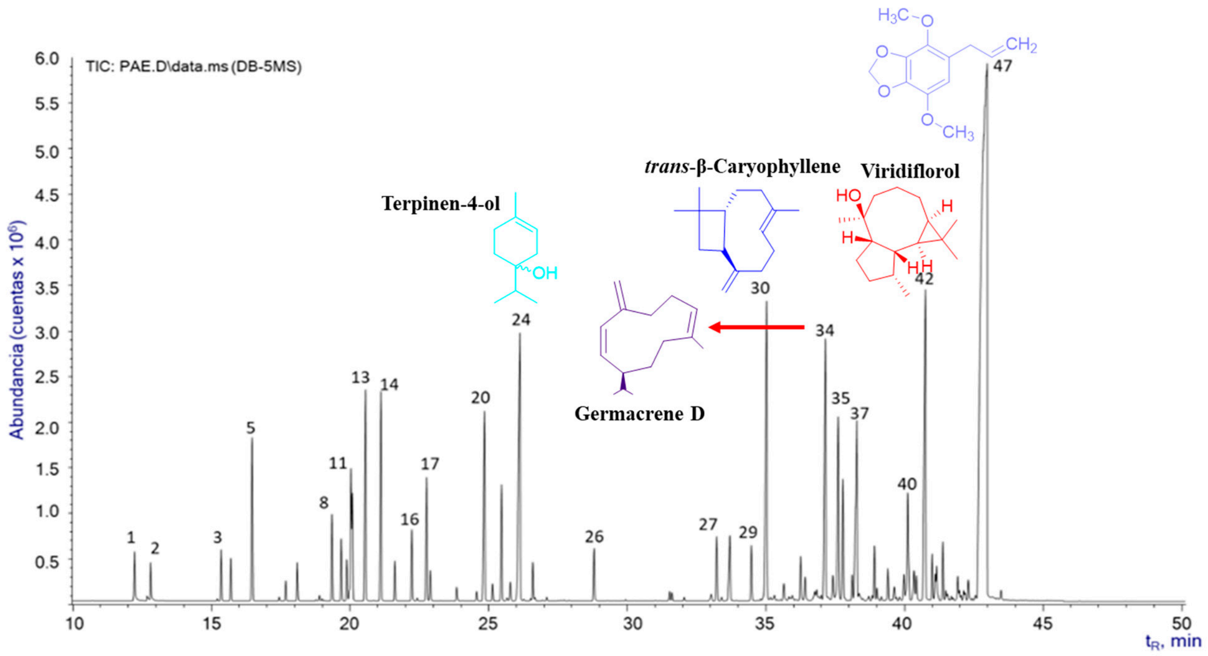

Figure S3. Chromatographic profile obtained by GC/MS (full scan) of the essential oil of *P. cumanense*. DB-5MS column (60 m), 1:30 split, MSD (EI, 70 eV).

Table S1. Treatments and inhibition percentages of the application of essential oils of *P. glabratum*, *P. friedrichsthalii*, and *P. cumanense* against *M. roreri* and *P. palmivora*.

Treatment	*M. roreri*					
	P. glabratum		*P. friedrichsthalii*		*P. cumanense*	
	concentration mg/mL	% inhibition	concentration mg/mL	% inhibition	concentration mg/mL	% inhibition
T1	4.44	56.00	18.7	69.26	5.3	24
T2	8.88	72.00	31.3	80.11	10.6	64
T3	44.4	86.00	114.7	90.78	52.3	100
T4	88.8	94.67	205.5	95.84	105.9	100
T5	440.5	100	639.9	100	524.2	100
T6 (PDA)	0.00	0.00	0.00	0.00	0.00	0.00
T7 control (+) (Copper oxychlorid e)	2.00	100	2.0	100	2.0	100
**Treatment**	*P. palmivora*					
	P. glabratum		*P. friedrichsthalii*		*P. cumanense*	
	concentration mg/mL	% inhibition	concentration mg/mL	% inhibition	concentration mg/mL	% inhibition
T1	4.44	2.00	18.7	24.33	5.3	10.00
T2	8.88	0.00	31.3	23.89	10.6	16.00
T3	44.4	5.3	114.7	39.78	52.3	76
T4	88.8	74.00	205.5	89.44	105.9	98
T5	440.5	100	639.9	100	524.2	100
T6 (Carrot-agar)	0.00	0.00	0.00	0.00	0.00	0.00
T7 control (+)(Metalax yl)	2.00	100	2.0	100	2.0	100

Table S2. Descriptive statistics and normality tests (Kolmogorov-Smirnov) for treatments applied to different *Piper* species against *M. roreri* and *P. palmivora*.

	M. roreri											
	P. glabratum				*P. friedrichsthalii*				*P. cumanense*			
Treatment	Average	Kolmogorov-Smirnov			Average	Kolmogorov-Smirnov			Average	Kolmogorov-Smirnov		
		Statistics	gl	Sig.		Statistics	gl	Sig.		Statistics	gl	Sig.
T1	0.81	0.385	3	1.000	0.51	0.175	3	0.000	1.5	0.377	3	0.000
T2	0.52	0.314	3	0.463	0.364	0.242	3	0.000	0.5	0.385	3	0.000
T3	0.24	0.292	3	0.363	0.24	0.333	3	0.000	0.000	0.000	3	0.000
T4	0.11	0.175	3	0.000	0.217	0.314	3	0.000	0.000	0.000	3	0.000
T5	0.00	0.000	3	0.000	0.000	0.000	3	0.000	0.000	0.000	3	0.000
T6	2.63	0.385	3	0.000	1.000	0.385	3	0.000	2.7	0.385	3	0.000
T7	0.00	0.000	3	0.000	0.000	0.000	3	0.000	0.000	0.000	3	0.000

	P. palmivora											
	P. glabratum				*P. friedrichsthalii*				*P. cumanense*			
Treatment	Average	Kolmogorov-Smirnov			Average	Kolmogorov-Smirnov			Average	Kolmogorov-Smirnov		
		Statistics	gl	Sig.		Statistics	gl	Sig.		Statistics	gl	Sig.
T1	4.90	0.245	3	0.000	2.2389	0.274	3	0.000	2.9	0.268	3	0.000
T2	5.00	0.385	3	0.000	0.2389	0.253	3	0.000	3.1	0.355	3	0.000
T3	4.73	0.227	3	0.000	1.6278	0.264	3	0.000	0.7	0.328	3	0.000
T4	1.30	0.221	3	0.000	0.9500	0.276	3	0.000	0.000	0.385	3	0.000
T5	0.000	0.000	3	0.000	0.0000	0.000	3	0.000	0.000	0.000	3	0.000
T6	5.00	0.257	3	0.000	2.4278	0.257	3	0.000	2.7	0.257	3	0.000
T7	0.000	0.000	3	0.000	0.0000	0.000	3	0.000	0.000	0.000	3	0.000

Table S3. Anova test of the essential oils of *P. glabratum*, *P. friedrichsthalii* and *P. cumanense* against *M. roreri* and *P. palmivora*.

	M. roreri																	
	P. glabratum						***P. friedrichsthalii***						***P. cumanense***					
	**Sum of squares**	**gl**	**Quadratic mean**	**F**	**Sig.**	**Eta squared**	**Sum of squares**	**gl**	**Quadratic mean**	**F**	**Sig.**	**Eta squared**	**Sum of squares**	**gl**	**Quadratic mean**	**F**	**Sig.**	**Eta squared**
**Intergroup**	6	6	2.636	140.360	0,000	0,9837	16.552	6	2.759	91.040	0.000	0,9750	18.610	6	3.102	267.361	0.000	0,9913
**Inside groups**	0.263	14	0.019				0.424	14	0.030				0.162	14	0.012			
**Total**	16.079	20					16.976	20					18.772	20				
	P. palmivora																	
	P. glabratum						***P. friedrichsthalii***						***P. cumanense***					
	**Sum of squares**	**gl**	**Quadratic mean**	**F**	**Sig.**	**Eta squared**	**Sum of squares**	**gl**	**Quadratic mean**	**F**	**Sig.**	**Eta squared**	**Sum of squares**	**gl**	**Quadratic mean**	**F**	**Sig.**	**Eta squared**
**Intergroup**	53.378	6	8.896	169.184	0.000	0.9864	19.548	6	3.258	59.093	0.000	0.9620	36.171	6	6.029	130.889	0.000	0.9825
**Inside groups**	0.736	14	0.053				0.772	14	0.055				0.645	14	0.046			
**Total**	54.114	20					20.320	20					36.816	20				

Results of ANOVA analysis to evaluate the efficacy of essential oils of *P. glabratum*, *P. friedrichsthalii* and *P. cumanense* against *M. roreri* and *P. palmivora*. The table shows the sum of squares, degrees of freedom (gl), root mean square, F-values and their significance (Sig.) for between-group and within-group effects in each treatment.

Table S4. Test of homogeneity of variances of the essential oil *P. glabratum*, *P. friedrichsthalii* and *P. cumanense* against *M. roreri* and *P. palmivora*.

		M. roreri											
		P. glabratum				***P. friedrichsthalii***				***P. cumanense***			
		**Levene's statistic**	**gl1**	**gl2**	**Sig.**	**Levene's statistic**	**gl1**	**gl2**	**Sig.**	**Levene's statistic**	**gl1**	**gl2**	**Sig.**
**Average**	**Based on the average**	8.81<br>1	6	14	0.000	5.520	6	14	0.000	13.338	6	14	0.000
		P. palmivora											
		P. glabratum				***P. friedrichsthalii***				***P. cumanense***			
		**Levene's statistic**	**gl1**	**gl2**	**Sig.**	**Levene's statistic**	**gl1**	**gl2**	**Sig.**	**Levene's statistic**	**gl1**	**gl2**	**Sig.**
**Average**	**Based on the average**	3.511	6	14	0.0025	4.631	6	14	0.009	5.679	6	14	0.004

Levene's test of homogeneity of variances to evaluate the variability of the effects of *P. glabratum*, *P. friedrichsthalii* and *P. cumanense* essential oil against *M. roreri* and *P. palmivora*. The values of Levene's statistic, the degrees of freedom (gl1 and gl2) and the significance (Sig.) based on the averages are reported.

Table S5. T2-Tamhane test comparisons of *P. glabratum* essential oil versus *M. roreri*.

Multiple comparisons

(I) Treatment	(J) Treatment	Difference of means (I-J)	Desv. error	Sig.	Confidence interval of 95%	
					Lower limit	Upper limit
T1	T2	0.28667*	0.03756	0.037	0.0228	0.5505
	T3	0.57000	0.15546	0.729	-2.1174	3.2574
	T4	0.70000*	0.03512	0.002	0.4301	0.9699
	T5	0.81000*	0.02887	0.026	0.2266	1.3934
	T6	-1.82333	0.13968	0.084	-4.1545	0.5078
	T7	0.81000*	0.02887	0.026	0.2266	1.3934
T2	T1	-0.28667*	0.03756	0.037	-0.5505	-0.0228
	T3	0.28333	0.15463	0.991	-2.5137	3.0803
	T4	0.41333*	0.03127	0.005	0.1936	0.6330
	T5	0.52333*	0.02404	0.043	0.0376	1.0091
	T6	-2.11000	0.13876	0.068	-4.5561	0.3361
	T7	0.52333*	0.02404	0.043	0.0376	1.0091
T3	T1	-0.57000	0.15546	0.729	-3.2574	2.1174
	T2	-0.28333	0.15463	0.991	-3.0803	2.5137
	T4	0.13000	0.15406	1.000	-2.7496	3.0096
	T5	0.24000	0.15275	0.998	-2.8470	3.3270
	T6	-2.39333*	0.20497	0.007	-3.8044	-0.9823
	T7	0.24000	0.15275	0.998	-2.8470	3.3270
T4	T1	-0.70000*	0.03512	0.002	-0.9699	-0.4301
	T2	-0.41333*	0.03127	0.005	-0.6330	-0.1936
	T3	-0.13000	0.15406	1.000	-3.0096	2.7496
	T5	0.11000	0.02000	0.489	-0.2942	0.5142
	T6	-2.52333	0.13812	0.050	-5.0576	0.0110
	T7	0.11000	0.02000	0.489	-0.2942	0.5142
T5	T1	-0.81000*	0.02887	0.026	-1.3934	-0.2266
	T2	-0.52333*	0.02404	0.043	-1.0091	-0.0376
	T3	-0.24000	0.15275	0.998	-3.3270	2.8470
	T4	-0.11000	0.02000	0.489	-0.5142	0.2942
	T6	-2.63333	0.13667	0.055	-5.3953	0.1286
	T7	0.00000	0.00000	0.00000	0.00000	0.00000
T6	T1	1.82333	0.13968	0.084	-0.5078	4.1545
	T2	2.11000	0.13876	0.068	-0.3361	4.5561
	T3	2.39333*	0.20497	0.007	0.9823	3.8044
	T4	2.52333	0.13812	0.050	-0.0110	5.0576
	T5	2.63333	0.13667	0.055	-0.1286	5.3953
	T7	2.63333	0.13667	0.055	-0.1286	5.3953
T7	T1	-0.81000*	0.02887	0.026	-1.3934	-0.2266
	T2	-0.52333*	0.02404	0.043	-1.0091	-0.0376
	T3	-0.24000	0.15275	0.998	-3.3270	2.8470
	T4	-0.11000	0.02000	0.489	-0.5142	0.2942
	T5	0.00000	0.00000	0.00000	0.00000	0.00000
	T6	-2.63333	0.13667	0.055	-5.3953	0.1286

*The difference in means is significant at the 0.05 level.

Table S6. T2-Tamhane test multiple comparisons of *P. glabratum* essential oil against *Phytophthora palmivora*.

Multiple comparisons

(I) Treatment	(J) Treatment	Difference of means (I-J)	Desv. error	Sig.	Confidence interval of 95%	
					Lower limit	Upper limit
T1	T2	-0.02333	0.18962	1.000	-3.0645	3.0178
	T3	0.77000	0.24824	0.544	-0.9373	2.4773
	T4	3.25433*	0.20455	0.014	1.1681	5.3406
	T5	3.78333*	0.18460	0.049	0.0527	7.5140
	T6	1.35333	0.29242	0.204	-0.7176	3.4243
	T7	3.78333*	0.18460	0.049	0.0527	7.5140
T2	T1	0.02333	0.18962	1.000	-3.0178	3.0645
	T3	0.79333	0.17153	0.518	-1.8312	3.4178
	T4	3.27767*	0.09818	0.002	2.3007	4.2546
	T5	3.80667*	0.04333	0.003	2.9309	4.6824
	T6	1.37667	0.23089	0.384	-2.6021	5.3554
	T7	3.80667*	0.04333	0.003	2.9309	4.6824
T3	T1	-0.77000	0.24824	0.544	-2.4773	0.9373
	T2	-0.79333	0.17153	0.518	-3.4178	1.8312
	T4	2.48433*	0.18790	0.018	0.7272	4.2415
	T5	3.01333	0.16597	0.062	-0.3407	6.3674
	T6	0.58333	0.28103	0.919	-1.5085	2.6752
	T7	3.01333	0.16597	0.062	-0.3407	6.3674
T4	T1	-3.25433*	0.20455	0.014	-5.3406	-1.1681
	T2	-3.27767*	0.09818	0.002	-4.2546	-2.3007
	T3	-2.48433*	0.18790	0.018	-4.2415	-0.7272
	T5	0.52900	0.08810	0.433	-1.2514	2.3094
	T6	-1.90100	0.24330	0.141	-4.8184	1.0164
	T7	0.52900	0.08810	0.433	-1.2514	2.3094
T5	T1	-3.78333*	0.18460	0.049	-7.5140	-0.0527
	T2	-3.80667*	0.04333	0.003	-4.6824	-2.9309
	T3	-3.01333	0.16597	0.062	-6.3674	0.3407
	T4	-0.52900	0.08810	0.433	-2.3094	1.2514
	T6	-2.43000	0.22679	0.166	-7.0132	2.1532
	T7	0.00000	0.00000	0.00000	0.00000	0.00000
T6	T1	-1.35333	0.29242	0.204	-3.4243	0.7176
	T2	-1.37667	0.23089	0.384	-5.3554	2.6021
	T3	-0.58333	0.28103	0.919	-2.6752	1.5085
	T4	1.90100	0.24330	0.141	-1.0164	4.8184
	T5	2.43000	0.22679	0.166	-2.1532	7.0132
	T7	2.43000	0.22679	0.166	-2.1532	7.0132
T7	T1	-3.78333*	0.18460	0.049	-7.5140	-0.0527
	T2	-3.80667*	0.04333	0.003	-4.6824	-2.9309
	T3	-3.01333	0.16597	0.062	-6.3674	0.3407
	T4	-0.52900	0.08810	0.433	-2.3094	1.2514
	T5	0.00000	0.00000	0.00000	0.00000	0.00000
	T6	-2.43000	0.22679	0.166	-7.0132	2.1532

*The difference in means is significant at the 0.05 level.

*The difference in means is significant at the 0.05 level.

Table S7. T2-Tamhane test multiple comparisons of the essential oil of *P. friedrichsthalii* versus *M. roreri*.

Multiple comparisons

(I) Treatment	(J) Treatment	Difference of means (I-J)	Desv. error	Sig.	Confidence interval of 95%	
					Lower limit	Upper limit
T1	T2	0.37143	0.14545	0.843	-1.0360	1.7788
	T3	0.67143	0.18535	0.632	-1.5832	2.9261
	T4	1.27143*	0.06950	0.024	0.3397	2.2032
	T5	1.32857	0.06598	0.050	-0.0049	2.6620
	T6	-1.30476	0.15305	0.081	-2.8642	0.2547
	T7	1.32857	0.06598	0.050	-0.0049	2.6620
T2	T1	-0.37143	0.14545	0.843	-1.7788	1.0360
	T3	0.30000	0.21634	0.997	-1.2912	1.8912
	T4	0.90000	0.13145	0.315	-1.4422	3.2422
	T5	0.95714	0.12963	0.315	-1.6625	3.5768
	T6	-1.67619*	0.18940	0.019	-2.9696	-0.3827
	T7	0.95714	0.12963	0.315	-1.6625	3.5768
T3	T1	-0.67143	0.18535	0.632	-2.9261	1.5832
	T2	-0.30000	0.21634	0.997	-1.8912	1.2912
	T4	0.60000	0.17457	0.792	-2.6813	3.8813
	T5	0.65714	0.17321	0.745	-2.8432	4.1575
	T6	-1.97619*	0.22152	0.023	-3.5580	-0.3943
	T7	0.65714	0.17321	0.745	-2.8432	4.1575
T4	T1	-1.27143*	0.06950	0.024	-2.2032	-0.3397
	T2	-0.90000	0.13145	0.315	-3.2422	1.4422
	T3	-0.60000	0.17457	0.792	-3.8813	2.6813
	T5	0.05714	0.02182	0.932	-0.3839	0.4981
	T6	-2.57619*	0.13981	0.048	-5.1028	-0.0496
	T7	0.05714	0.02182	0.932	-0.3839	0.4981
T5	T1	-1.32857	0.06598	0.050	-2.6620	0.0049
	T2	-0.95714	0.12963	0.315	-3.5768	1.6625
	T3	-0.65714	0.17321	0.745	-4.1575	2.8432
	T4	-0.05714	0.02182	0.932	-0.4981	0.3839
	T6	-2.63333	0.13810	0.056	-5.4241	0.1575
	T7	0.00000	0.00000	0.00000	0.00000	0.00000
T6	T1	1.30476	0.15305	0.081	-0.2547	2.8642
	T2	1.67619*	0.18940	0.019	0.3827	2.9696
	T3	1.97619*	0.22152	0.023	0.3943	3.5580
	T4	2.57619*	0.13981	0.048	0.0496	5.1028
	T5	2.63333	0.13810	0.056	-0.1575	5.4241
	T7	2.63333	0.13810	0.056	-0.1575	5.4241
T7	T1	-1.32857	0.06598	0.050	-2.6620	0.0049
	T2	-0.95714	0.12963	0.315	-3.5768	1.6625
	T3	-0.65714	0.17321	0.745	-4.1575	2.8432
	T4	-0.05714	0.02182	0.932	-0.4981	0.3839
	T5	0.00000	0.00000	0.00000	0.00000	0.00000
	T6	-2.63333	0.13810	0.056	-5.4241	0.1575

*The difference in means is significant at the 0.05 level.

Table S8. T2-Tamhane test multiple comparisons of the essential oil of *P. friedrichsthalii* versus *P. palmivora.*

(I)Treatment	(J) Treatment	Difference of means (I-J)	Desv. error	Sig.	Confidence interval of 95%	
					Lower limit	Upper limit
T1	T2	2.00000	0.15416	0.109	-0.9858	4.9858
	T3	0.61111	0.26954	0.875	-1.4610	2.6833
	T4	1.28889	0.16842	0.118	-0.5057	3.0834
	T5	2.23889	0.15346	0.094	-0.8624	5.3402
	T6	-0.18889	0.27296	1.000	-2.3119	1.9341
	T7	2.23889	0.15346	0.094	-0.8624	5.3402
T2	T1	-2.00000	0.15416	0.109	-4.9858	0.9858
	T3	-1.38889	0.22208	0.401	-5.7855	3.0077
	T4	-0.71111	0.07093	0.143	-1.8937	0.4714
	T5	0.23889	0.01470	0.076	-0.0582	0.5359
	T6	-2.18889	0.22621	0.194	-6.6706	2.2928
	T7	0.23889	0.01470	0.076	-0.0582	0.5359
T3	T1	-0.61111	0.26954	0.875	-2.6833	1.4610
	T2	1.38889	0.22208	0.401	-3.0077	5.7855
	T4	0.67778	0.23221	0.831	-2.5431	3.8986
	T5	1.62778	0.22160	0.318	-2.8505	6.1061
	T6	-0.80000	0.31633	0.755	-2.9526	1.3526
	T7	1.62778	0.22160	0.318	-2.8505	6.1061
T4	T1	-1.28889	0.16842	0.118	-3.0834	0.5057
	T2	0.71111	0.07093	0.143	-0.4714	1.8937
	T3	-0.67778	0,23221	0.831	-3.8986	2.5431
	T5	0.95000	0.06939	0.105	-0.4523	2.3523
	T6	-1.47778	0.23616	0.286	-4.7899	1.8343
	T7	0.9500	0.06939	0.105	-0.4523	2.3523
T5	T1	-2.23889	0.15346	0.094	-5.3402	0.8624
	T2	-0.23889	0.01470	0.076	-0.5359	0.0582
	T3	-1.62778	0.22160	0.318	-6.1061	2.8505
	T4	-0.95000	0.06939	0.105	-2.3523	0.4523
	T6	-2.42778	0.22574	0.165	-6.9897	2.1342
	T7	0.00000	0.00000	0.00000	0.00000	0.00000
T6	T1	0.18889	0.27296	1.000	-1.9341	2.3119
	T2	2.18889	0.22621	0.194	-2.2928	6.6706
	T3	0.80000	0.31633	0.755	-1.3526	2.9526
	T4	1.47778	0.23616	0.286	-1.8343	4.7899
	T5	2.42778	0.22574	0.165	-2.1342	6.9897
	T7	2.42778	0.22574	0.165	-2.1342	6.9897
T7	T1	-2.23889	0.15346	0.094	-5.3402	0.8624
	T2	-0.23889	0.01470	0.076	-0.5359	0.0582
	T3	-1.62778	0.22160	0.318	-6.1061	2.8505
	T4	-0.95000	0.06939	0.105	-2.3523	0.4523
	T5	0.00000	0.00000	0.00000	0.00000	0.00000
	T6	-2.42778	0.22574	0.165	-6.9897	2.1342

* The difference in means is significant at the 0.05 level.

Table S9. T2-Tamhane test multiple comparisons of the essential oil of *P. cumanense* (p) versus *M. roreri*

(I) Treatment	(J) Treatment	Difference of means (I-J)	Desv. Error	Sig.	Confidence interval of 95% Lower limit	Upper limit
T1	T2	0.84048	0.08944	0.206	-0.9430	2.6239
	T3	1.40714	0.08931	0.081	-0.3977	3.2120
	T4	1.40714	0.08931	0.081	-0.3977	3.2120
	T5	1.40714	0.08931	0.081	-0.3977	3.2120
	T6	-1.22619	0.16446	0.064	-2.5487	0.0963
	T7	1.40714	0.08931	0.081	-0.3977	3.2120
T2	T1	-0.84048	0.08944	0.206	-2.6239	0.9430
	T3	0.56667*	0.00476	0.001	0.4704	0.6629
	T4	0.56667*	0.00476	0.001	0.4704	0.6629
	T5	0.56667*	0.00476	0.001	0.4704	0.6629
	T6	-2.06667	0.13818	0.088	-4.8435	0.7102
	T7	0.56667*	0.00476	0.001	0.4704	0.6629
T3	T1	-1.40714	0.08931	0.081	-3.2120	0.3977
	T2	-0.56667*	0.00476	0.001	-0.6629	-0.4704
	T4	0.00000	0.00000	0.000	0.0000	0.0000
	T5	0.00000	0.00000	0.000	0.0000	0.0000
	T6	-2.63333	0.13810	0.056	-5.4241	0.1575
	T7	0.00000	0.00000	0.000	0.0000	0.0000
T4	T1	-1.40714	0.08931	0.081	-3.2120	0.3977
	T2	-0.056667*	0.00476	0.001	-0.6629	-0.4704
	T3	0.00000	0.00000	0.000	0.0000	0.0000
	T5	0.00000	0.00000	0.000	0.0000	0.0000
	T6	-2.63333	0.13810	0.056	-5.4241	0.1575
	T7	0.00000	0.00000	0.000	0.0000	0.0000
T5	T1	-1.40714	0.08931	0.081	-3.2120	0.3977
	T2	-0.56667*	0.00476	0.001	-0.6629	-0.4704
	T3	0.00000	0.00000	0.000	0.0000	0.0000
	T4	0.00000	0.00000	0.000	0.0000	0.0000
	T6	-2.63333	0.13810	0.056	-5.4241	0.1575
	T7	0.00000	0.00000	0.000	0.0000	0.0000
T6	T1	1.22619	0.16446	0.064	-0.0963	2.5487
	T2	2.06667	0.13818	0.088	-0.7102	4.8435
	T3	2.63333	0.13810	0.056	-0.1575	5.4241
	T4	2.63333	0.13810	0.056	-0.1575	5.4241
	T5	2.63333	0.13810	0.056	-0.1575	5.4241
	T7	2.63333	0.13810	0.056	-0.1575	5.4241
T7	T1	-1.40714	0.08931	0.081	-3.2120	0.3977
	T2	-0.56667*	0.00476	0.001	-0.6629	-0.4704
	T3	0.00000	0.00000	0.000	0.0000	0.0000
	T4	0.00000	0.00000	0.000	0.0000	0.0000
	T5	0.00000	0.00000	0.000	0.0000	0.0000
	T6	-2.63333	0.13810	0.056	-5.4241	0.1575

* The difference in means is significant at the 0.05 level.

Table S10. T2-Tamhane test multiple comparisons of the essential oil of *P. cumanense* (P) versus *P. palmivora*

(I) Tratamiento	(J) Tratamiento	Diferencia de medias (I-J)	Desv. Error	Sig.	Intervalo de confianza al 95%	
					Límite inferior	Límite superior
T1	T2	-0.012778	0.23544	1.000	-1.7323	1.4768
	T3	2.12222	0.16574	0.092	-0.7386	4.9831
	T4	2.86667	0.16320	0.063	-0.3604	6.0938
	T5	2.89444	0.16283	0.064	-0.3961	6.1850
	T6	0.46667	0.27833	0.983	-1.6219	2.5552
	T7	2.89444	0.16283	0.064	-0.3961	6.1850
T2	T1	0.12778	0.23544	1.000	-1.4768	1.7323
	T3	2.25000	0.17285	0.091	-0.7701	5.2701
	T4	2.99444	0.17042	0.063	-0.3814	6.3703
	T5	3.02222	0.17006	0.064	-0.4145	6.4590
	T6	0.59444	0.28262	0.910	-1.4773	2.6662
	T7	3.02222	0.17006	0.064	-0.4145	6.4590
T3	T1	-2.12222	0.16574	0.092	-4.9831	0.7386
	T2	-2.25000	0.17285	0.091	-5.2701	0.7701
	T4	0.74444*	0.03287	0.012	0.3275	1.1614
	T5	0.77222*	0.03093	0.033	0.1471	1.3973
	T6	-1.65556	0.22785	0.297	-5.8841	2.5730
	T7	0.77222*	0.03093	0.033	0.1471	1.3973
T4	T1	-2.86667	0.16320	0.063	-6.0938	0.3604
	T2	-2.99444	0.17042	0.063	-6.3703	0.3814
	T3	-0.74444*	0.03287	0.012	-1.1614	-0.3275
	T5	0.02778	0.01111	0.946	-0.1968	0.2523
	T6	-2.40000	0.22601	0.166	-6.9157	2.1157
	T7	0.02778	0.01111	0.946	-0.1968	0.2523
T5	T1	-2.89444	0.16283	0.064	-6.1850	0.3961
	T2	-3.02222	0.17006	0.064	-6.4590	0.4145
	T3	-0.77222*	0.03093	0.033	-1.3973	-0.1471
	T4	-0.02778	0.01111	0.946	-0.2523	0.1968
	T6	-2.42778	0.22574	0.165	-6.9897	2.1342
	T7	0.00000	0.00000	0.000	0.0000	0.0000
T6	T1	-0.46667	0.27833	0.983	-2.5552	1.6219
	T2	-0.59444	0.28262	0.910	-2.6662	1.4773
	T3	1.65556	0.22785	0.297	-2.5730	5.8841
	T4	2.40000	0.22601	0.166	-2.1157	6.9157
	T5	2.42778	0.22574	0.165	-2.1342	6.9897
	T7	2.42778	0.22574	0.165	-2.1342	6.9897
T7	T1	-2.89444	0.16283	0.064	-6.1850	0.3961
	T2	-3.02222	0.17006	0.064	-6.4590	0.4145
	T3	-0.77222*	0.03093	0.033	-1.3973	-0.1471
	T4	-0.02778	0.01111	0.946	-0.2523	0.1968
	T5	0.00000	0.00000	0.000	0.0000	0.0000
	T6	-2.42778	0.22574	0.165	-6.9897	2.1342

* The difference in means is significant at the 0.05 level.

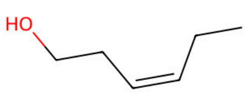

1) cis-Hex-3-en-1-ol

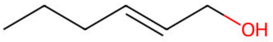

2) trans-Hex-2-en-1-ol

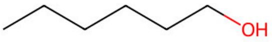

3) Hexan-1-ol

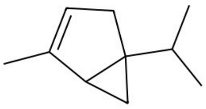

4) Alpha-Thujene

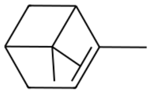

5) Alpha-Pinene

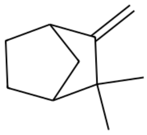

6) Camphene

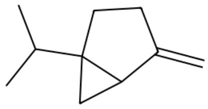

7) Sabinene

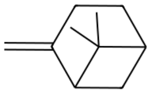

8) Beta-Pinene

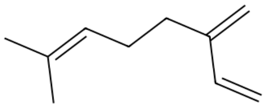

9) Beta-Myrcene

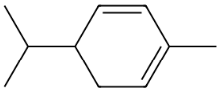

10) Alpha-Phellandrene

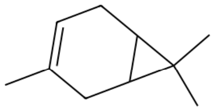

11) Delta3-Carene

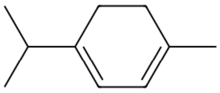

12) Alpha-Terpinene

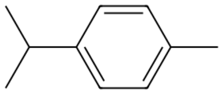

13) p-Cimene

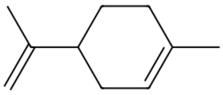

14) Limonene

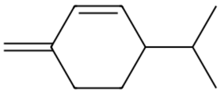

15) Beta-Phellandreno

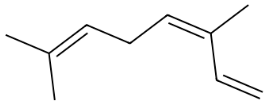

16) cis-Beta-Ocimene

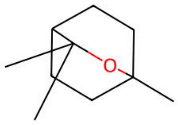

17) 1,8-Cineol

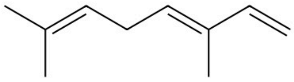

18) trans-Beta-Ocimene

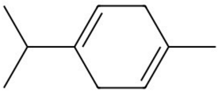

19) Gamma-Terpinene

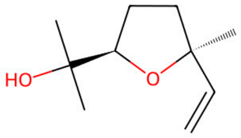

20) cis-Linalool oxide

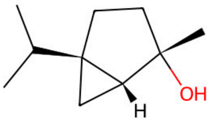

21) cis-Sabinene hydrate

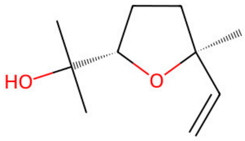

22) trans-Linalool oxide

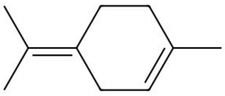
23) Terpinolene

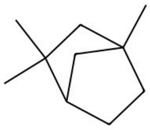
24) Fenchone

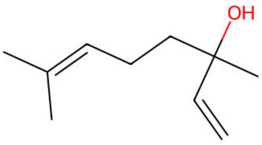

25) Linalool

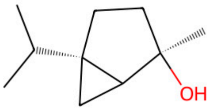

26) trans-Sabinene hydrate

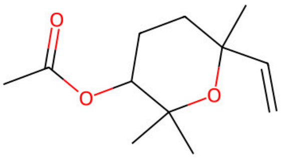

27) 2,2,6-Trimethyl-3-keto-6-vinyl-tetrahydropyran

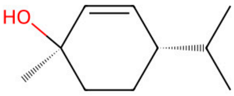

28) Trans-p-Ment-2-en-1-ol

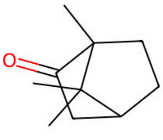

29) Camphor

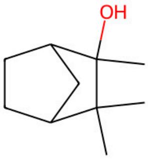

30) Camphene hydrate

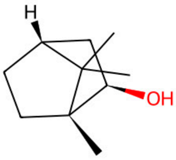

31) Isoborneol

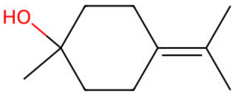

32) Delta-Terpineol

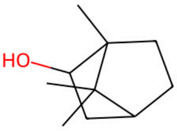

33) Borneol

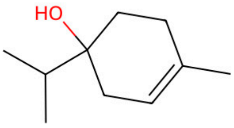

34) Terpinen-4-ol

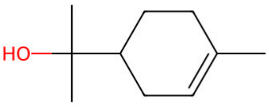

35) Alpha-Terpineol

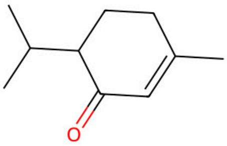

36) Piperitone

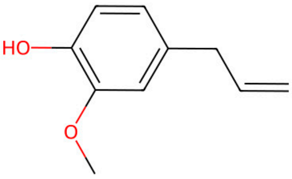

37) Eugenol

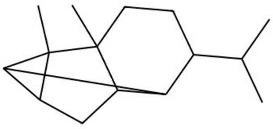
38) Cyclosativene

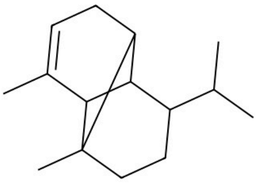
39) Alpha-Copaene

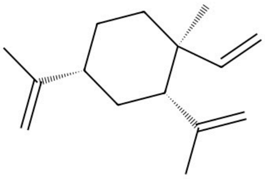
40) Beta-Elemene

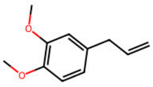

41) Methyleugenol

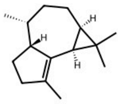

42) Alpha-Gurjunene

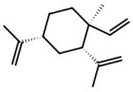

43) Beta-Elemene

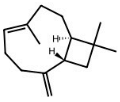

44) trans-Beta-Caryophyllene

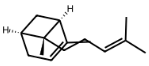

45) trans-Alpha-Bergamotene

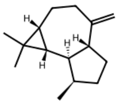

46) Aromadendrene

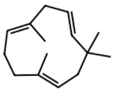

47) Alpha-Humuleno

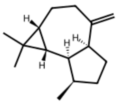

48) Alloaromadendreno

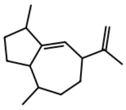

49) Gamma-Gurjunene

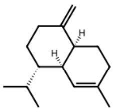

50) Gamma-Muurolene

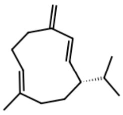

51) Germacrene D

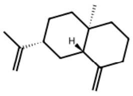

52) Beta-Selinene

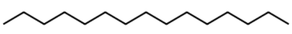

53) Pentadecane

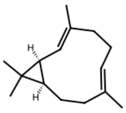

54) Bicyclogermacrene

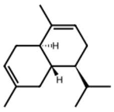

55) Cadin-3,9-diene

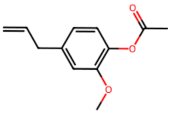

56) Eugenyl acetate

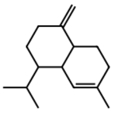

57) Gamma-Cadinene

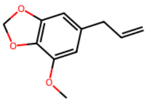

58) Myristicin

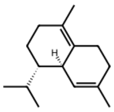

59) Delta-Cadinene

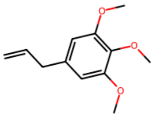

60) Elemicin

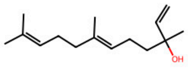

61) trans-Nerolidol

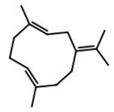

62) Germacrene B

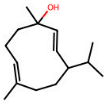

63) Germacrene D-4-ol

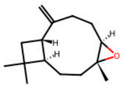

64) Caryophyllene oxide

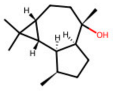

65) Viridiflorol

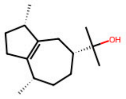

66) Guaiol

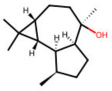

67) Ledol

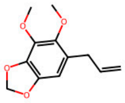

68) Dil-apiole

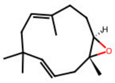

69) Humulene epoxide II

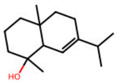

70) Selin-6-en-4Alpha-ol

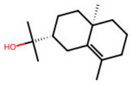

71) Gamma-Eudesmol

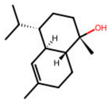

72) epi-Alpha-Cadinol

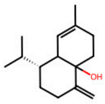

73) Murola-4-en-10-ol

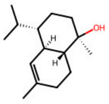

74) Alpha-Cadinol

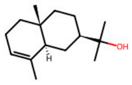

75) Alpha-Eudesmol

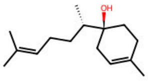

76) Beta-Bisabolol

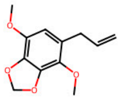

77) Apiole

78) Heptadecane

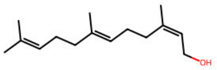

79) 2-cis-6-trans-Farnesol

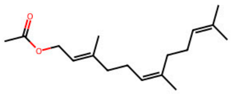

80) 2-cis-6-trans-farnesyl acetate

Figure S4. 2D chemical structure of compounds identified in the essential oils of the species *P. glabratum, P. friedrichsthalii, and P. cumanense*.
